# A Single Amino Acid Able to Promote High‐Temperature Ring‐Opening Polymerization by Dual Activation

**DOI:** 10.1002/advs.202308956

**Published:** 2024-02-13

**Authors:** Elena Gabirondo, Katarzyna Świderek, Edurne Marin, Ainhoa Maiz‐Iginitz, Aitor Larranaga, Vicent Moliner, Agustin Etxeberria, Haritz Sardon

**Affiliations:** ^1^ POLYMAT Department of Advanced Polymers and Materials: Physics, Chemistry and Technology Faculty of Chemistry University of the Basque Country UPV/EHU Manuel de Lardizabal 3 Pasealekua Donostia 20018 Spain; ^2^ BioComp Group Institute of Advanced Materials (INAM), Universitat Jaume I Castelló 12071 Spain; ^3^ Department of Mining‐Metallurgy Engineering and Materials Science, POLYMAT Faculty of Engineering in Bilbao University of the Basque Country (UPV/EHU) Plaza Torres Quevedo 1 Bilbao 48013 Spain; ^4^ Center for Cooperative Research in Biomaterials (CIC biomaGUNE) Basque Research and Technology Alliance (BRTA) Paseo de Miramon 182 San Sebastián Spain

**Keywords:** biocompatible, enzyme‐like mechanism, organocatalysis, ring‐opening polymerization, taurine

## Abstract

Amino acids are indispensable compounds in the body, performing several biological processes that enable proper functioning. In this work, it is demonstrated that a single amino acid, taurine, is also able to promote the ring‐opening polymerization (ROP) of several cyclic monomers under industrially relevant conditions. It is shown that the unique zwitterionic structure of taurine, where the negatively charged sulfonic acid group and the protonated amine group are separated by two methylene groups, not only provides high thermal stability but also leads to a dual activation mechanism, which is corroborated by quantum mechanical calculations. This unique mechanism allows for the synthesis of polylactide of up to 50 kDa in bulk at 180 °C with good end‐group fidelity using a highly abundant catalyst. Furthermore, cytotoxicity tests confirm that PLLA synthesized with taurine is non‐toxic. Moreover, it is demonstrated that the presence of taurine does not have any detrimental effect on the thermal stability of polylactide, and therefore polymers can be used directly without any post‐polymerization purification. It is believed that the demonstration that a simple structure composed of a single amino acid can promote polymerization can bring a paradigm shift in the preparation of polymers.

## Graphical abstract

Taurine is a 100% natural, biocompatible, and thermally stable compound. This work demonstrates that it can be used as a catalyst for the ring‐opening polymerization of cyclic compounds, while maintaining stereoregularity. In addition, its lack of cytotoxicity and detrimental effect on the thermal stability of polymers can make it a suitable catalyst for sustainable polymerization processes.

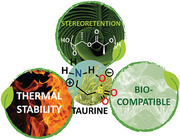

## 1 Introduction

Although catalysts are typically used in relatively small quantities in the manufacture of polymers, they are a key component that enables the design of energy‐efficient polymerization processes. The use of catalysis makes it possible to improve operating conditions in terms of pressure, time, and/or temperature, with consequent energy savings. Finding new and better catalysts is therefore of great importance in order to reduce the carbon footprint of polymer products.^[^ ^1^, ^2^ ^]^ Traditionally biocatalysis and metal‐catalysis have been primarily used in industrial systems, but over the last 20 years organocatalytic methods, which make use of low‐molecular‐weight organic catalysts, have been developed that can be as efficient and selective as metal‐ and biocatalytic pathways.^[^ ^3^, ^4^, ^5^, ^6^ ^]^

The main benefit of organocatalysts as compared to organometallic catalysts resides in their versatility, high selectivity, and the possibility of purification or recovery of the catalyst from the final polymer. However, to date, the majority of industrial processes are based on metal catalysts such as tin (II) octoate and titanium (IV) tetrabutoxide.^[^ ^7^ ^]^ In some applications, the use of certain metals such as tin is problematic due to strict regulations that restrict their use in several products, and alternatives that are less toxic and more biocompatible are desired. While the use of organocatalysts can mitigate some of these drawbacks, in some industrial processes such as the Ring‐Opening Polymerization of L‐Lactide or the step‐growth polymerization of poly(ethylene terephthalate) elevated temperatures are usually employed to improve the polymerization kinetics.

As many organic compounds possess relatively poor thermal stability, high temperatures can lead to the degradation of the catalyst during polymerization. Our group, among others, has recently demonstrated that one strategy to address these thermal stability issues is the use of organocatalysts based on hydrogen bond donor–acceptor adducts.^[^ ^8^, ^9^, ^10^, ^11^, ^12^, ^13^, ^14^ ^]^ These catalysts have recently been proven to be stable and active at elevated temperatures, in some cases at temperatures close to 400 °C. Using this concept, the ring‐opening polymerization (ROP) of L‐Lactide (L‐LA) at elevated temperatures as well as some polycondensation reactions have been performed with great success.^[^ ^15^, ^16^, ^17^, ^18^ ^]^

**Figure 1:**
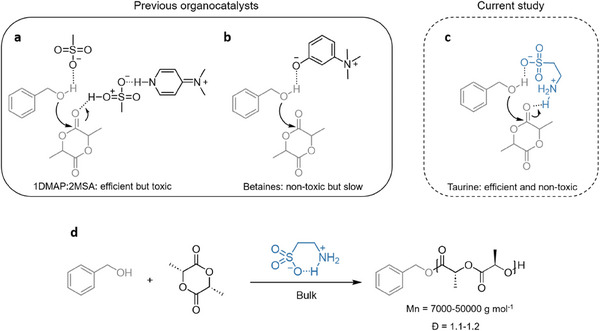
Taurine, is a naturally occurring catalyst for high‐temperature ring‐opening polymerizations.

As improved thermal stability is mainly the result of strong ionic interactions, sulfonic acids tend to provide higher thermal stability.^[^ ^8^, ^18^, ^19^, ^20^ ^]^ However, commercially available sulfonic acids such as methanesulfonic acid (MSA) have some important limitations including their relatively high toxicity, which prevents their use in applications related to the food industry,^[^ ^21^ ^]^ and/or the need to use an inert atmosphere due to their strong interaction with water. One common approach to mitigate the toxicity issues is to replace synthetic organocatalysts with naturally occurring catalysts with similar functions. For instance, Guo et al. have found that by using a saccharin‐based catalytic system the ROP of L‐LA could be promoted.^[^ ^22^ ^]^ Similarly, Coulembier et al. investigated the use of naturally occurring ammonium betaines for the ROP of L‐LA and cyclic carbonates at room temperature in solution, obtaining rapid polymerizations.^[^ ^23^ ^]^ While several naturally occurring catalysts have been investigated, as far as we are aware none of them are competitive in terms of performance with other non‐natural organocatalysts and their ability to operate efficiently in industrially relevant polymerization conditions has not been explored (**Figure** **1**a,b).

Among the bio compounds with a sulfonic group in their structure, in this work, we look at the use of taurine (2‐aminoethanesulfonic acid). This compound is present in the tissues of humans and animals, thus demonstrating its high biocompatibility. Taurine has several functions in the human body including its involvement in osmoregulation, and its behavior as an antioxidant and immunomodulator. It is also thought that taurine acts as a neuroprotector, as it is one of the most abundant free amino acids in the brain.^[^ ^24^, ^25^ ^]^ Very recently, the ability of taurine as a green bio‐organic catalyst in the promotion of some organic transformations such as the Knoevenagel condensation between aromatic aldehydes and malononitrile has been demonstrated.^[^ ^26^ ^]^ Taurine acts as a bifunctional donor–acceptor reagent in which the carbonyl site of L‐lactide is activated by taurine and then attacked by the negatively activated alcohol group of benzyl alcohol (Figure 1c).

In this work, we investigate the use of taurine for the high‐temperature polymerization of cyclic esters, with a particular focus on the homopolymerization of L‐lactide (Figure 1d). We initially hypothesized that despite the presence of the highly nucleophilic primary amine, this compound could potentially be a suitable catalyst as taurine only exists in the form of zwitterion even in water. Furthermore, due to its conformational arrangement, it was thought that taurine would present comparable thermal stability to other donor–acceptor type catalysts, such as MSA‐based salts. To corroborate our initial hypothesis we first compare the efficiency of taurine to the highly efficient catalyst formed by the mixture of 4‐(dimethylamino)‐pyridine (DMAP) and MSA, DMAP:MSA, in the bulk polymerization of lactide.^[^ ^27^, ^28^, ^29^ ^]^ After demonstrating the comparable behavior of taurine with respect to DMAP:MSA, we provide some experimental and theoretical evidence of the dual activation mechanism of taurine. We also evaluate the potential of taurine to mediate other types of ROP processes and its potential to mediate the preparation of block copolymers. Finally, we explore the toxicity of taurine and compare it to alternative acidic and basic organocatalysts.

## 2 Results and Discussion

### 2.1 Screening of Taurine as a Catalyst for the ROP of L‐LA

**Figure 2:**
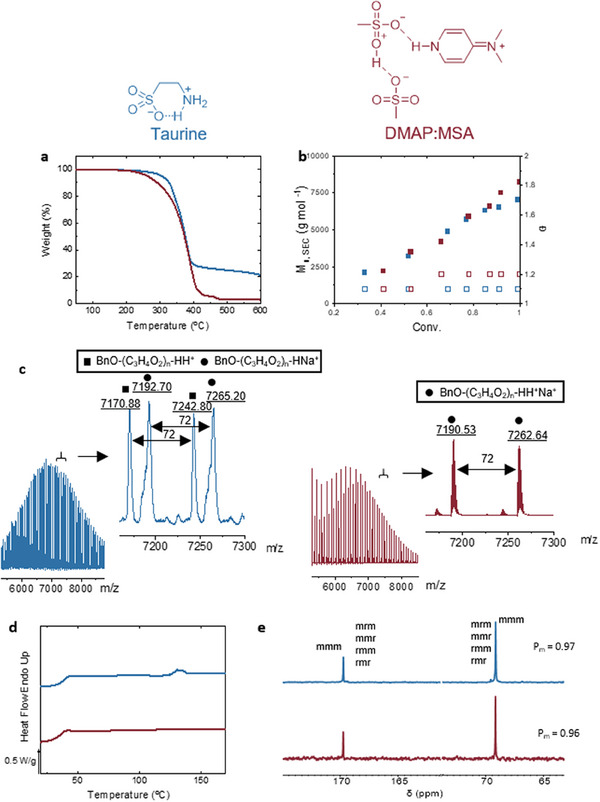
Comparison between DMAP:MSA (1:2) and taurine. a) TGA analysis, b) M_n_ evolution (solid squares) and the dispersity (non‐solid squares) of the polymerization, c) MALDI‐TOF analysis, d) DSC analysis, and e) ^13^C NMR spectrum and P_m_ values. Results obtained with DMAP:MSA (1:2) are shown in red while those with taurine are in blue.

Although previous work demonstrating the potential use of amino acid derivatives as catalysts for polymerization reactions has been reported, in all cases the attained results have not been competitive when compared to polymerizations involving non‐natural catalysts.^[^ ^23^, ^30^, ^31^ ^]^ In order to evaluate the potential of taurine as an activator for the bulk ROP of lactide, we first evaluated the thermal stability of taurine by thermogravimetric analysis (TGA), and the results were compared to the DMAP:MSA base–acid mixture that has previously been shown to be an efficient catalyst for the ROP of L‐LA at high temperatures (**Figure** **2**a). Taurine showed excellent thermal stability and was stable up to 250–300 °C, thus displaying improved stability when compared to DMAP:MSA, which started degrading at 200 °C. Taking advantage of the high thermal stability, we explored the role of taurine as an activator for the bulk ROP of L‐lactide at 180 °C (Figure 1d and **Table** **1**). 5 mol.% of taurine was mixed with benzyl alcohol (BnOH) and L‐LA to obtain target DP values ([LA]_0_/[BnOH]_0_) between 50 and 400, as shown in Table 1.

**Table 1:** Conditions and results for the ring‐opening polymerization of L‐lactide in bulk, initiated with benzyl alcohol.

Entry	Catalyst	[LA]_0_/[BnOH]_0_	Temp. [°C]	Time [h]	Conv. [%]	M_n,theo_ [g mol^−1^]	M_n,SEC_ [g mol^−1^]^a)^	Ð	T_m_ [°C]
1	DMAP:MSA	50	180	1	98	7200	9000	1.2	–
2	Taurine	50	180	4	99	7200	7000	1.1	137.6
3	Taurine	50	160	48	98	7200	6900	1.1	148.3
4	Taurine	50	130	96	95	7200	6500	1.1	140.8
5	Taurine	100	180	7	98	14 200	14 100	1.1	–
6	Taurine	150	180	10	97	21 000	20 500	1.2	–
7	Taurine	400	180	24	97	56 000	50 400	1.2	–
8^b)^	Taurine	50	180	4	98	5700	7200	1.2	–
9^c)^	Taurine	50	180	4	94	5100	5300	1.3	–
10	Tin octoate	50	180	3	99	7200	7500	1.2	152.3

^a)^ Determined by SEC in THF with polystyrene standards and correction factors;

^b)^ ROP of ε‐caprolactone;

^c)^ ROP of trimethylene carbonate.

This procedure led to controlled ROP of L‐LA to afford poly(lactides) (PLAs) with molecular weights that conform to those predicted theoretically (M_n,theo_) from the ratio [LA]_0_/[BnOH]_0_ and the measured conversion. Moreover, the molecular weight distribution in all cases was narrow (Đ = 1.1–1.2) suggesting a living‐like polymerization process and a lack of transesterification. In order to corroborate both the living character and the lack of transesterification, the molecular weight and dispersity evolution were examined (Figure 2b). A linear trend of M_n,SEC_ with conversion was observed, confirming a living polymerization character in the presence of taurine. Finally, MALDI‐TOF analysis was performed to demonstrate the end‐group fidelity (Figure 2c). The separation of the peaks was 72 m/z, which corresponds to the lactyl unit of the lactide, with two different signals, the polymer chain plus Na^+^ and the polymer chain plus H^+^ with no signals present that could be attributed to direct initiation by taurine. While the molecular weights are predictable, the separation of 72 m/z between peaks could indicate the formation of PLA cycles by back‐biting reaction or some transesterification reactions.^[^ ^32^ ^]^ In conclusion, it could be confirmed that the polymerization of L‐LA by taurine is a controlled polymerization and the behavior is similar to that when using DMAP:MSA catalyst.

One of the challenges in the ROP of L‐LA is to avoid side reactions such as transesterification and epimerization which leads to non‐stereoregular PLA. The control of the polymer microstructure is of great importance since it affects the mechanical and thermal properties of the obtained PLA. In order to evaluate if taurine could provide the same degree of control of DMAP:MSA ^13^C NMR, DSC, and SEC were performed for different polymerization degrees (50, 100, 150, and 400) at 180 °C in bulk (Figure 2d; Figure S1, Supporting Information). Analysis of the resulting poly(L‐lactide) by ^13^C NMR and DSC revealed the presence of atactic sequences and a melting point of 138 °C only in the case of DP = 50 (Figure 2d,e). The rest of the polymers with higher DP values were completely amorphous (Table 1, entries 5, 6, and 7; Figure S1, Supporting Information). It should be pointed out that these values surpassed the behavior of DMAP:MSA but were not sufficiently good to provide PLLA with decent crystallinity degrees. Tin octoate, an industrially used metal‐based catalyst, was also tried, with similar molecular weights and dispersities observed, but with an increase in Tm up to 152.3 °C (Table 1, entry 10; Figure S2, Supporting Information).

**Figure 3:**
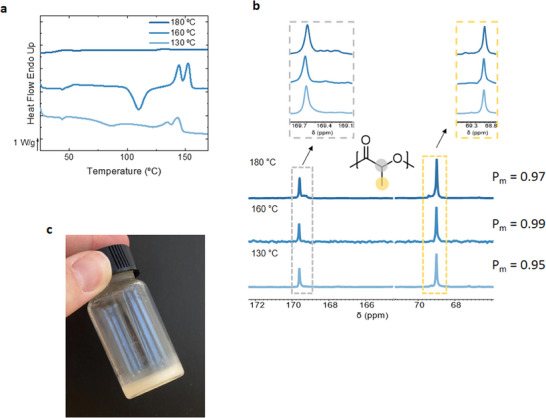
Comparison of the crystallinity of PLA synthesized using different temperatures by a) DSC analysis, b) ^13^C NMR and P_m_ values, and c) image of the final polymer.

In our previous studies, we have shown that temperature could play a major role in the preparation of isotactic PLA.^[^ ^18^ ^]^ Indeed, when performing the ROP of L‐LA at temperatures lower than 160 °C, the polymerization led to semicrystalline PLA (**Figure** **3**a), confirming the reduction of epimerization at lower reaction temperatures. These results are in good agreement with the result observed in the ^13^C NMR analysis where it is seen that there is a reduction of epimerization with the reduction in temperature (Figure 3b). Further decrease of temperature to 130 °C did not provide any improvement in the crystallization of PLA. We speculate that high activation energies (as later supported by the computational studies) prevented reaching the desired molecular weight, thus resulting in a decrease in the melting temperature (from 148.3 in entry 3 to 140.8 °C in entry 4). From all these results it could be concluded that taurine is an effective catalyst for the synthesis of PLA by ROP of L‐LA. In order to evaluate if the specific structure of taurine is important or if it is sufficient to have a salt based on a sulfonic acid and a primary amine to mediate the polymerization of L‐LA, we decided to perform a model reaction using methanesulfonic acid:butylamine salt. Unfortunately, by using this salt the ROP of L‐LA resulted in a very low molecular weight PLA polymer. ^1^H NMR spectroscopy showed that butylamine was also acting as an initiator in the polymerization (Figure S3, Supporting Information). Similarly, two unimolecular acid‐base compounds, L‐proline and creatine were also used for the polymerization of L‐LA (Figure S4, Supporting Information). SEC curves indicated that the Mn of the PLA synthesized by the use of two catalysts was lower than expected. In order to confirm the initiator character they were mixed with L‐Lactide without the addition of an extra initiator and the initiation mediated by the L‐proline as creatine was verified by ^1^H NMR spectra (Figure S4, Supporting Information).

### 2.2 In Silico Investigation of the ROP of the L‐LA Mechanism

For the understanding of the mechanism of the polymerization of L‐LA by taurine, ^13^C NMR study was carried out by putting in contact L‐LA and taurine for one side and BnOH and taurine for the other side (Figures S5 and S6, Supporting Information). In the case of BnOH and taurine (Figure S6, Supporting Information), it was observed that the signals were shifting, indicating an interaction between them, however, there was no observed interaction between L‐LA and taurine (Figure S5, Supporting Information). We believe that this interaction could be more pronounced at elevated temperatures and in solvent‐free conditions.

In order to get some insights into the mechanism of polymerization of L‐LA using taurine, a comprehensive computational study was conducted. Initial models of the reactive complexes and isolated molecules were prepared and optimized at the density functional theory (DFT) level employing the long‐range corrected hybrid density functional with damped atom‐atom dispersion corrections ωB97XD^[^ ^33^, ^34^ ^]^ and the global hybrid functional with 54% HF exchange M06‐2X.^[^ ^35^ ^]^ Before the catalytic mechanism was explored, the influence of the environment on the protonation states of the taurine, as well as on butylamine‐methane sulfonic acid salt (BuNH_2_:MSA), both used in experiments as catalysts, was studied. Potential energy surfaces (PESs) were computed for the intramolecular proton exchange between the amino and sulfone groups present in taurine as well as for intermolecular proton transfer from the amino (─NH_2_) group of BuNH_2_ to the sulfone (─SO_3_) group of MSA in the gas phase and in condensed media (Figure S7, Supporting Information). A significant difference in relative stabilization energies was found for the structures depending on the environment. While in the gas phase, both neutral variants of the taurine molecule and BuNH_2_:MSA complex are thermodynamically more stable, in a model imitating more realistic conditions of the process (in solution), the zwitterionic/ionic pair appears to be more favorable. Higher stabilization of the charged species of catalysts in comparison to their neutral form was confirmed by results obtained using the two different functionals. The observed deviations in the results obtained in the gas phase and condensed media highlighted the significant role of the environment in the studied process and forced us to include its presence in the rest of the calculations. X‐ray structure confirmed that the taurine exists in the form of a zwitterion as the sulfonic acid group is negatively charged, and the amine group is fully protonated and positively charged (Figure S8, Supporting Information).

**Figure 4:**
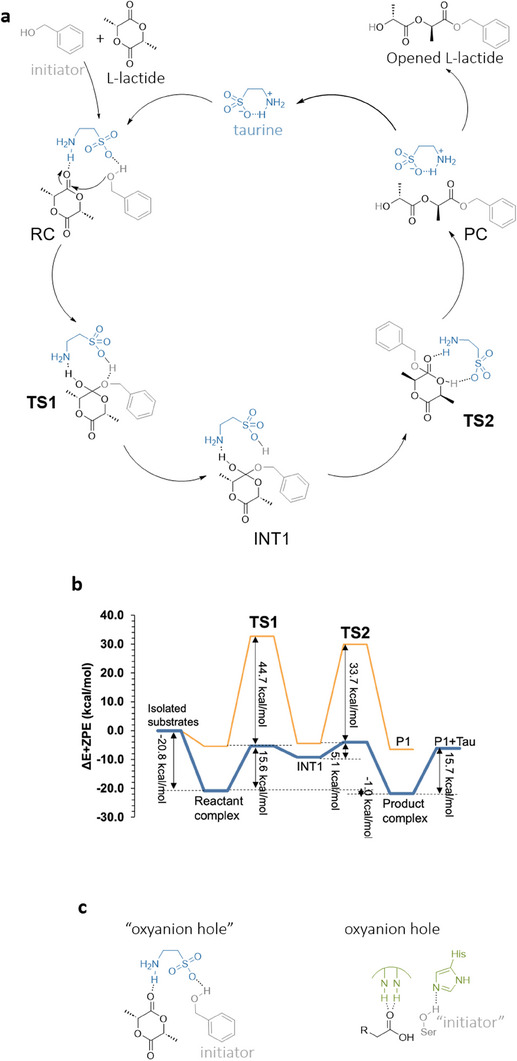
Mechanism of polymerization of L‐LA using taurine. a) Reaction mechanism for taurine‐assisted initiation step of ring‐opening polymerization of L‐lactide(L‐LA) with benzyl alcohol (BnOH) initiator. b) Energy profile for the uncatalyzed (orange line) and taurine‐assisted ring‐opening polymerization of L‐lactide(L‐LA) with benzyl alcohol (BnOH) initiator (blue line) computed at M06‐2X/6‐31+G(d,p) level in an ethyl lactate solvent at T = 403 K. c) Schematic comparison between the geometrical preorganization of an enzyme active site (i.e., that of the CALB in depolymerizing PET)^[^ ^38^ ^]^ versus taurine.

After evaluating the structure of taurine in solution, the mechanisms and kinetics of the ROP process for L‐LA with BnOH in the presence of taurine as a catalyst were studied and compared to the uncatalyzed ROP process (Figure S9, Supporting Information). The initiation step in the presence of a zwitterionic form of taurine, as a catalyst, was explored starting from computing the energy of reactant complex formation (ΔE + ZPE_f_). As shown in **Figure** **4**a, the complex formed between taurine, L‐LA, and BnOH (Tau:L‐LA:BnOH) is more stable (by 8.4 or 6.0 kcal mol^−1^, depending on the functional) than the complex formed by two charged taurine molecules, confirming that the formation of the reactant complex is thermodynamically feasible and favorable (Figure 4b). In the taurine:L‐LA:BnOH case, taurine forms strong and specific hydrogen bond (H‐bond) interactions between the amino group and the carbonyl oxygen of L‐LA (electrophilic activation), while the sulfone group interacts with the hydroxyl group of BnOH (nucleophilic activation). The established H‐bond interaction between reactants and catalyst ensures the reactive orientation required for the first step of the reaction, that is the nucleophilic attack, by bringing the nucleophilic group and the electrophilic center close together (see Supporting Information for details). Thus, the mechanism of the ROP determined computationally in the presence of taurine takes place in two steps where the ring‐opening of L‐LA is preceded by the nucleophilic attack of BnOH to the carbonyl carbon (C═O^L‐LA^) of L‐LA. The main difference between the uncatalyzed process (Figure S10, Supporting Information) and the mechanism assisted by taurine originates in the alternative shuffling of hydrogen atoms during the reaction (Figure 4a). Therefore, instead of being transferred directly to the oxygen of the carbonyl group of L‐LA (O═C^L‐LA^), the proton of the hydroxyl group of BnOH (H^BnOH^) is transferred to the oxygen of the sulfone group of taurine (O^Tau^) in the first step, while the negative charge accumulated on the oxygen of the carbonyl group during the reaction is neutralized by an additional proton (H^Tau^) that is transferred directly from the positively charged amino group of taurine, see Figure 4a. Such redirection for one proton transfer and the involvement of an additional proton in the process results in less restrained geometries of TS structures and, consequently, in a meaningful reduction of the energy barriers for the first step (up to 20 kcal mol^−1^ regarding the uncatalyzed process) as shown in Figure 4b. In the second step, the proton from the carbonyl oxygen (O═C^L‐LA^) is returned to the nitrogen (N^Tau^) of the amino group of taurine, while the proton located in the sulfone group is transferred to the oxygen (O^L‐LA(ring)^) of the L‐LA ring, resulting in the ester bond breaking and the opening of the ring (Figure 4a).

The obtained molecular mechanism agrees with the dual catalytic activation of lactide proposed by Waymouth, Hedricks, and co‐workers, in ROP.^[^ ^12^, ^36^, ^37^ ^]^ In addition, and despite the huge differences between this single aminoacid catalyst and the gigantic protein scaffolds, the specific groups present in taurine ensure a structural preorganization that is reminiscent of some of the features usually observed in the active sites of enzymes. Thus, the established H‐bond interactions between reactants and catalyst stabilize a reactive orientation in the reactants state non‐covalent complex, which reminds the preorganization concept of enzyme mechanisms as responsible for the reduction in the activation‐free energies. In addition, the stabilization of the charge developed on carbonyl oxygen atoms by interactions with the amino group resembles the presence of oxyanion holes in many enzymes, including, for instance, Candida antarctica Lipase B (CALB). According to our calculations, the structure of taurine provides the perfect position of the weak base, the ─SO_3_ group, and an acid/oxyanion hole, mimicked by the ─NH_3_ group. This perfect geometrical fit observed in the complex of taurine with both reactants (L‐LA and BnOH) is achieved due to the ideal two‐carbon “linker” present between the two catalytically active groups of taurine. The structure present in taurine thus plays a key role in providing a unique catalytic center to promote the polymerization of L‐LA, mimicking the dual activation behavior observed, for instance, in the depolymerization of PET by CALB (Figure 4c^[^ ^36^ ^]^).

Besides computing the mechanism in the presence of taurine we also evaluated the initiation step of the ROP process of L‐LA with BnOH assisted by BuNH_2_:MSA salt in its ionic form, as an alternative catalyst to taurine (Figure S11, Supporting Information). It was found that the order of atom redistribution along the reaction according to the revealed molecular mechanism is identical to the one observed in the taurine‐assisted process, although in the case of BuNH_2_:MSA the two catalytically active groups are provided by separate molecules. As shown in Figure S12A (Supporting Information), the activation energy barrier increases up to 19.4 kcal mol^−1^ in ROP when the BuNH_2_:MSA salt is used (vs the r.d.s. of 16.8 kcal mol^−1^ in the taurine‐assisted process). In addition, the product complex was found to be slightly less stable (by 0.6 kcal mol^−1^) than the reactant complex. A comparative analysis of the evolution of the charges along the reaction suggests that the lower energy barrier of the first step in the reaction with taurine can be associated with a lower reorganization of the electron density, especially in the case of the O^Tau^ versus the O^Salt^ from RC to TS1 (−0.690 to −0.620 a.u. vs −0.750 to −0.558 a.u. in the case of taurine and butylamine‐methane sulfonic acid salt, respectively). The intrinsic barrier of the second step, measured from the intermediate, is in fact lower when using the salt than when using taurine (Figure 4b vs Figure S12A, Supporting Information). Once again, the higher catalytic activity of taurine can be related to an electrostatic pre‐organization, resembling that observed in enzymes.

Besides reducing the rate of the primary process, it is also possible that the use of BuNH_2_:MSA salt as a catalyst can also induce a secondary reaction where the BuNH_2_ plays the role of initiator. Taking this possibility into account, the alternative variant of the reaction was computationally explored and the influence of both initiators on the rate of the ROP process was analyzed. Surprisingly, the computed energy profile, as shown in Figure S12B (Supporting Information), revealed that the “secondary” process is kinetically more favorable, which is demonstrated by a notably lower activation energy barrier (15.3 kcal mol^−1^) than the one computed for the same process with BnOH as initiator. Furthermore, the obtained product complex proved to be more stable (by 9.9 kcal mol^−1^) than the reactant complex, and therefore the product of the reaction between BuNH_2_ and L‐LA is thermodynamically more favorable. This computational finding is in excellent agreement with experimental results demonstrating that when BuNH_2_:MSA salt is used in the ROP of L‐LA, the amine can directly react with L‐LA. It is worth mentioning that the “secondary” reaction is rather unfeasible in the case of taurine because, as demonstrated computationally, this molecule is present only in its ionized form, therefore preventing the formation of the highly reactive neutral amino group. Finally, computational and experimental work proves that the use of taurine unequivocally has an advantage over salt in catalyzing the ROP process of L‐LA with BnOH.

### 2.3 Extending the Catalytic Scope of Taurine

It has been demonstrated that taurine is an effective catalyst for the ROP of L‐lactide. To demonstrate its versatility, we investigated the potential of taurine to promote the bulk polymerization of other cyclic lactones and carbonate monomers such as ε‐caprolactone (CL) or trimethylene carbonate (TMC) (Table 1 Entry 8 and 9). We found that the polymerizations were slower than those of lactide but exhibited exceptional control over the polymerization, with predictable molecular weights, end group fidelity, and narrow molecular weight distributions (Table 1 entries 8 and 9; Figure S13a,b, Supporting Information). Moreover, taurine was also used as a catalyst in the synthesis of well‐defined diblock copolymers of PLA‐b‐PCL by sequential addition of lactide and ε‐caprolactone (Figure S13c, Supporting Information). First, the polymerization of L‐LA was performed, using the same reaction conditions as described previously, resulting in a polymer with a molecular weight of 7000 g·mol^−1^ and a dispersity of 1.1. After that, ε‐caprolactone was added to the reaction mixture. It was confirmed by GPC that the reaction proceeded, with an increase in the molecular weight up to 13 900 g mol^−1^ whilst maintaining the dispersity of 1.1 (Figure S13c, Supporting Information). In addition, the DSC of the block copolymer was also performed and compared to PLA and PCL homopolymers, observing at ≈55 °C the Tm value of PCL and ≈137 °C the one of PLA (Figure S13c, Supporting Information). These results demonstrate the potential of taurine to act as a catalyst for the living polymerization of cyclic esters and lactones.

### 2.4 Toxicity of Taurine

As mentioned previously, taurine is a naturally occurring catalyst that is found in many animals and has attracted growing interest because it is considered to be a sustainable compound. In addition, as it is present in animal tissues, it is thought to be non‐toxic. To confirm the lack of toxicity of taurine, PLA from Entry 2, Table 1 was analyzed as received without any purification. To explore the potential biocompatibility of taurine‐based PLA, the cytotoxicity of the material was assessed using two different PLA‐taurine extracts: 100% (i.e., the extract obtained from the incubation of the material in a complete medium at 37 °C for 24 h) and 10% (i.e., a 1:10 dilution of the 100% extract). The metabolic activity of HeLa cells in the presence of the taurine‐based PLA extract was measured after 24 and 72 h by means of the Alamar Blue assay and compared to other two PLAs obtained with conventional catalysts: a basic one (i.e., 1,8‐diazabicyclo(5.4.0)undec‐7‐ene (DBU)) and an acidic one (i.e., methanesulfonic acid (MSA)). Cells incubated with complete medium and complete medium with 10% DMSO were considered negative and positive controls, respectively.

**Figure 5:**
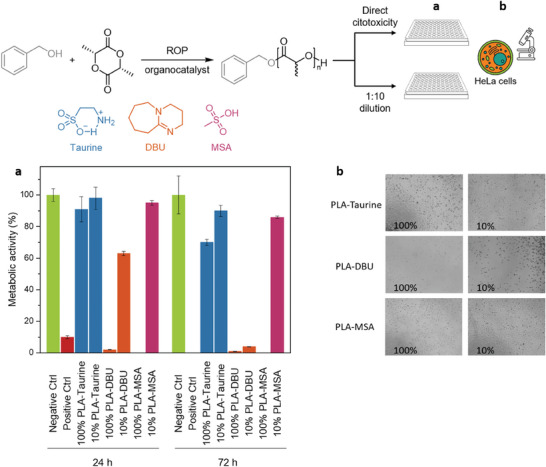
Toxicity test of PLLA synthesized with taurine. a) Cytotoxicity test of taurine and its comparison to MSA and DBU and b) the optical microscope images.

When PLA‐DBU (i.e., polylactide synthesized using DBU as a catalyst) and PLA‐MSA (i.e., polylactide synthesized using MSA as a catalyst) were placed in contact with the culture medium, an instantaneous color change to yellow was observed, indicating that these two polymers induce a significant change of the pH of the complete medium. In contrast, in the case of PLA‐taurine (i.e., polylactide synthesized using taurine as a catalyst) no color change was observed in the medium. Furthermore, the cells were able to maintain the metabolic activity above the threshold value (i.e., 70%) in the presence of the PLA‐taurine extracts, at both extract concentrations (i.e., 10%, and 100%) (**Figure** **5**a). These results indicate no toxic effects of PLA‐taurine at any of the concentrations tested. The polymer synthesized using DBU as a catalyst induced a significant decrease in the metabolic activity of the cells in the case of 10% extract concentration at 24 h, while in the presence of concentrated extract (i.e., 100%), the activity of the cells was reduced to almost zero (Figure 5a). In the case of PLA‐MSA at 100% concentration, the metabolic activity was null. However, at the diluted concentration (i.e., 10%), the metabolic activity was maintained ≈80%, indicating that PLA‐MSA is not as toxic as PLA‐DBU (Figure 5a).

These results agreed with the observations by optical microscopy. In the case of PLA‐taurine and PLA‐MSA, a regular cell density and distribution were observed in the presence of 10% extract concentration (Figure 5b). When the extract concentration was increased to 100%, fewer cells were observed using PLA‐MSA and only a few cells showed a round shape in the presence of PLA‐taurine (Figure 5b), suggesting minimal cytotoxic response. In the presence of PLA‐DBU at both concentrations, the cells showed a round shape, which is characteristic of non‐viable cells (Figure 5b). Therefore, the polylactide that is synthesized with 5% taurine shows the lowest toxicity among the studied compounds and could be used without the need for post‐polymerization purification.

**Figure 6:**
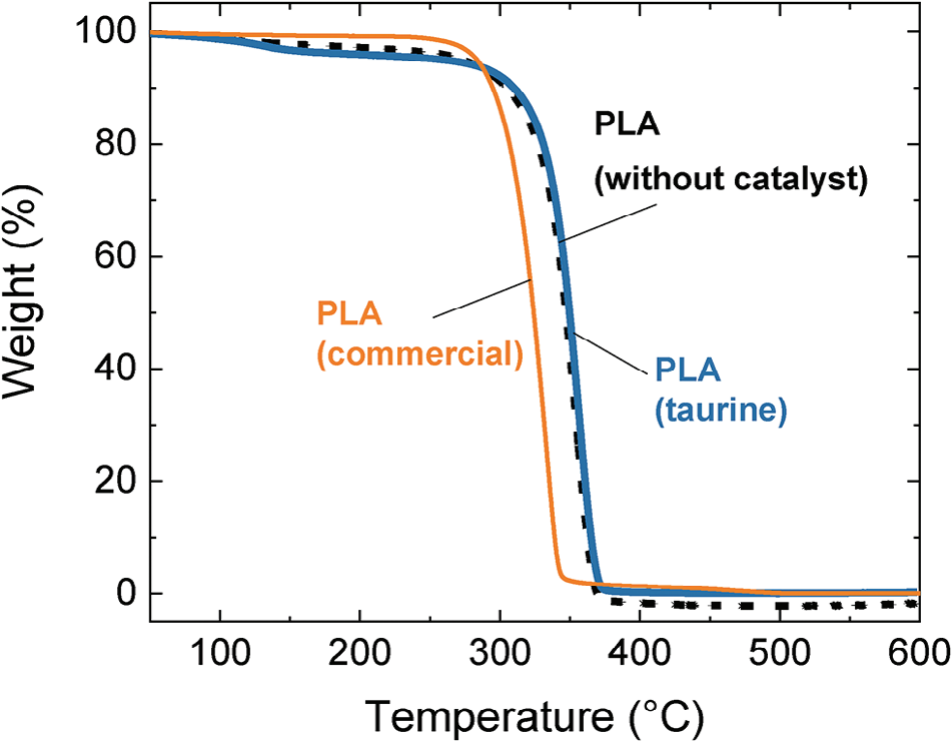
Comparison of TGA curves of PLA synthesized using taurine as a catalyst and without any purification (blue line), after purification (black line), and commercial PLA (orange line).

As taurine does not show any toxicity, in terms of biocompatibility its removal is not required. However, several works have reported that PLA polymer thermal stability could be detrimentally affected by traces of catalyst that could be present in the material.^[^ ^39^ ^]^ To investigate the influence that taurine could have on the thermal stability of PLA, TGA analyses were performed with and without a catalyst, and the results were compared with commercial PLA of 70 kDa (**Figure** **6**). As expected, the presence of the catalyst in the commercial PLA induces a negative impact on the thermal stability of PLA reducing considerably the thermal stability. Surprisingly we found that the presence of taurine does not affect the thermal stability of PLA. In addition, the hydrolytic depolymerization of PLA has been tried in the presence of taurine, in order to see if it has any negative effect in this process. It has been concluded, that PLA depolymerized into lactic acid at 160 °C and lactic acid and taurine were recovered (Figure S16, Supporting Information). These results indicate that taurine does not interfere negatively with the hydrolytic depolymerization of the polymer. These results together with the negligible cytotoxicity suggest that taurine is an excellent candidate for the polymerization of different cyclic monomers.

In summary, we report a highly abundant, biocompatible, and highly efficient amino acid for the bulk ROP of cyclic lactones and carbonates in industrial‐relevant conditions. The key advantage of this process is that it does not require any time‐consuming and costly removal of the catalyst from the final polymer as taurine is biocompatible and present in many tissues in animals. Computational studies indicate that the unique zwitterionic structure of taurine not only provides extremely high thermal stability but also allows the simultaneous activation of the alcohol initiator/chain end and the lactone monomer without providing any undesirable initiation, as the dual catalytic activation of lactide previously suggested by Hedrick and co‐workers. Surprisingly, the high selectivity of these catalysts for propagation over transesterification leads to stereocontrol in the polymerization of L‐LA as well as excellent end‐group fidelity as demonstrated by MALDI‐TOF analysis. More generally we expect that the use of taurine may have a potential impact in other industrially relevant polymerization processes to produce C─N and C─O containing polymers where there is a need to utilize high temperatures and where nucleophiles and electrophiles must be activated simultaneously.

## Conflict of Interest

The authors declare no conflict of interest.

## Supporting information

Supporting Information

## Data Availability

The data that support the findings of this study are available from the corresponding author upon reasonable request.

## References

[1] G. Ertl , Angew. Chem., Int. Ed. 2009, 48, 6600. 10.1002/anie.200901193 19536798

[2] E. Roduner , Chem. Soc. Rev. 2014, 43, 8226. 25311156 10.1039/c4cs00210e

[3] E. Gabirondo , A. Sangroniz , A. Etxeberria , S. Torres‐Giner , H. Sardon , Polym. Chem. 2020, 11, 4861.

[4] S. C. Pan , B. List , Ernst Schering Found Symp Proc 2007, 2, 1. 18646300

[5] N. E. Kamber , W. Jeong , R. M. Waymouth , R. C. Pratt , B. G. G. Lohmeijer , J. L. Hedrick , Chem. Rev. 2007, 107, 5813. 17988157 10.1021/cr068415b

[6] F. Nederberg , E. Connor , M. Möller , T. , Glauser , J. L. Hedrick , Angew. Chem., Int. Ed. 2001, 40, 2712. 10.1002/1521-3773(20010716)40:14<2712::AID-ANIE2712>3.0.CO;2-Z 29712329

[7] R. E. Drumright , P. R. Gruber , D. E. Henton , Adv. Mater. 2000, 12, 1841.

[8] A. Basterretxea , E. Gabirondo , C. Jehanno , H. Zhu , I. Flores , A. J. Müller , A. Etxeberria , D. Mecerreyes , O. Coulembier , H. Sardon , ACS Sustainable Chem. Eng. 2019, 7, 4103.

[9] C. Jehanno , I. Flores , A. P. Dove , A. J. Müller , F. Ruipérez , H. Sardon , Green Chem. 2018, 20, 1205.

[10] I. Flores , J. Demarteau , A. J. Müller , A. Etxeberria , L. Irusta , F. Bergman , C. Koning , H. Sardon , Eur. Polym. J. 2018, 104, 170.

[11] D. Delcroix , A. Couffin , N. Susperregui , C. Navarro , L. Maron , B. Martin‐Vaca , D. Bourissou , Polym. Chem. 2011, 2, 2249.

[12] D. J. Coady , K. Fukushima , H. W. Horn , J. E. Rice , J. L. Hedrick , Chem. Commun. 2011, 47, 3105. 10.1039/c0cc03987j 21258732

[13] Y. Hoashi , T. Okino , Y. Takemoto , Angew. Chem., Int. Ed. 2005, 44, 4032. 10.1002/anie.200500459 15906403

[14] J. Leclaire , M. Mazari , Y. Zhang , C. Bonduelle , O. Thillaye Du Boullay , B. Martin‐Vaca , D. Bourissou , I. De Riggi , R. Fortrie , F. Fotiadu , G. Buono , Chem. ‐ Eur. J. 2013, 19, 11301. 23832831 10.1002/chem.201301275

[15] J. Kadota , D. Pavlović , H. Hirano , A. Okada , Y. Agari , B. Bibal , A. Deffieux , F. Peruch , RSC Adv. 2014, 4, 14725.

[16] A. Basterretxea , E. Gabirondo , A. Sanchez‐Sanchez , A. Etxeberria , O. Coulembier , D. Mecerreyes , H. Sardon , Eur. Polym. J. 2017, 95, 650.

[17] S. Zhang , V. Lemaire , A. Féret , H. Lefebvre , M. Tessier , A. Fradet , Polym. Chem. 2013, 4, 1538.

[18] A. Basterretxea , E. Gabirondo , C. Jehanno , H. Zhu , O. Coulembier , D. Mecerreyes , H. Sardon , Macromolecules 2021, 54, 6214. 35693113 10.1021/acs.macromol.1c01060 PMC9171820

[19] M. Palenzuela , K. Sarisuta , M. Navarro , N. Kumamoto , N. Chanthaset , J. Monot , H. Ajiro , B. Martín‐Vaca , D. Bourissou , Macromolecules 2023, 56, 678.

[20] F. Kayser , G. Fleury , S. Thongkham , C. Navarro , B. Martin‐Vaca , D. Bourissou , Polym. Chem. 2022, 13, 2201.

[21] M. D. Gernon , M. Wu , T. Buszta , P. Janney , Green Chem. 1999, 1, 127.

[22] F. Wei , H. Zhu , Z. Li , H. Wang , Y. Zhu , L. Zhang , Z. Yao , Z. Luo , C. Zhang , K. Guo , Adv. Synth. Catal. 2019, 361, 1335.

[23] B. Guillerm , V. Lemaur , J. Cornil , R. Lazzaroni , P. Dubois , O. Coulembier , Chem. Commun. 2014, 50, 10098. 10.1039/c4cc03347g 25050414

[24] T. Bouckenooghe , C. Remacle , B. Reusens , Curr. Opin. Clin. Nutr. Metab. Care 2006, 9, 728. 17053427 10.1097/01.mco.0000247469.26414.55

[25] H. Ripps , W. Shen , Mol. Vision 2012, 18, 2673. PMC3501277 23170060

[26] F. Shirini , N. Daneshvar , RSC Adv. 2016, 6, 110190.

[27] S. R. Rogers , M. E. Long , T. E. Turner , Introduction to Synthetic Methods in Step‐Growth Polymers, 1st ed., John Willey & Sons, Hoboken, NJ 2003.

[28] P. J. Flory , Principles of Polymer Chemistry, 1st ed., Cornell University Press, Ithaca, NY, 1995.

[29] G. Odian , Principles of Polymerization, 4th ed., John Willey & Sons, Inc, Hoboken, NJ 2004.

[30] S. Kusumoto , S. Ito , K. Nozaki , Asian J. Org. Chem. 2013, 2, 977.

[31] E. Oledzka , M. Sobczak , M. Kolakowski , B. Kraska , W. Kamysz , W. Kolodziejski , Macromol. Res. 2013, 21, 161.

[32] O. Coulembier , J. De Winter , T. Josse , L. Mespouille , P. Gerbaux , P. Dubois , Polym. Chem. 2014, 5, 2103.

[33] J. D. Chai , M. Head‐Gordon , J. Chem. Phys. 2008, 128, 084106. 18315032 10.1063/1.2834918

[34] J. D. Chai , M. Head‐Gordon , Phys. Chem. Chem. Phys. 2008, 10, 6615. 18989472 10.1039/b810189b

[35] Y. Zhao , N. E. Schultz , D. G. Truhlar , J. Chem. Theory Comput. 2006, 2, 364. 26626525 10.1021/ct0502763

[36] M. K. Kiesewetter , E. J. Shin , J. L. Hedrick , R. M. Waymouth , Macromolecules 2010, 43, 2093.

[37] L. Zhang , R. C. Pratt , F. Nederberg , H. W. Horn , J. E. Rice , R. M. Waymouth , C. G. Wade , J. L. Hedrick , Macromolecules 2010, 43, 1660.

[38] K. Świderek , S. Velasco‐Lozano , M. À. Galmés , I. Olazabal , H. Sardon , F. López‐Gallego , V. Moliner , Nat. Commun. 2023, 14, 3556. 37321996 10.1038/s41467-023-39201-1 PMC10272158

[39] E. Wojtczak , P. Kubisa , M. Bednarek , Polym. Degrad. Stab. 2018, 151, 100.

