# Supplementary material for: A Single Amino Acid Able to Promote High‐Temperature Ring‐Opening Polymerization by Dual Activation
Source: Adv Sci (Weinh). 2024 Feb 13;11(16):2308956. doi: 10.1002/advs.202308956 (PMC11040373; doi:10.1002/advs.202308956)
Supplement: Supplementary file 1 — Supporting Information [file ADVS-11-2308956-s001.pdf]

ADVANCED SCIENCE
Open Access

Supporting Information

for *Adv. Sci.*, DOI 10.1002/advs.202308956

A Single Amino Acid Able to Promote High-Temperature Ring-Opening Polymerization by Dual Activation

*Elena Gabirondo, Katarzyna Świderek, Edurne Marin, Ainhoa Maiz-Iginitz, Aitor Larranaga, Vicent Moliner, Agustin Etxeberria and Haritz Sardon**

A single amino acid able to promote high-temperature ring-opening polymerization by dual activation

Elena Gabirondo[1], Katarzyna Świderek[2], Edurne Marin[3], Ainhoa Maiz-Iginitz[1,4], Aitor Larranaga[3], Vicent Moliner[2], Agustin Etxeberria[1] and Haritz Sardon[1,*]

[1] POLYMAT, Department of Advanced Polymers and Materials: Physics, Chemistry and Technology, Faculty of Chemistry, University of the Basque Country UPV/EHU, Manuel de Lardizabal 3 Pasealekua, 20018 Donostia, Spain

[2] BioComp Group, Institute of Advanced Materials (INAM), Universitat Jaume I, 12071 Castelló, Spain

[3] University of the Basque Country (UPV/EHU), Department of Mining-Metallurgy Engineering and Materials Science, POLYMAT, Faculty of Engineering in Bilbao, Plaza Torres Quevedo 1, 48013 Bilbao, Spain

[4] Center for Cooperative Research in Biomaterials (CIC biomaGUNE), Basque Research and Technology Alliance (BRTA), Paseo de Miramon 182, San Sebastián, Spain

Materials S2

Methods S2

^{1}H and [13]C Nuclear Magnetic Resonance (NMR) S2

Differential Scanning Calorimetry (DSC) S2

Thermogravimetric analysis (TGA) S2

Matrix Assisted Laser Desorption Ionization - Time of Flight (MALDI-TOF) analysis S3

Size Exclusion Chromatography (SEC) S3

X-ray diffraction S3

Toxicity test S3

Computational methods S3

Ring-opening polymerization of L-lactide S4

Ring-opening polymerization of ε-caprolactone S5

Ring-opening polymerization of trimethylene carbonate S5

Characterization Data and Results S6

References S17

Materials

Taurine (99 %, Sigma-Aldrich S.A.), 4-dimethylaminopyridine (DMAP) (99 %, TCI), methanesulfonic acid (MSA) (99 %, Sigma-Aldrich S.A.), 1,8-diazabicyclo(5.4.0)undec-7-ene (DBU) (98 %, Sigma-Aldrich S.A.), butylamine (99.5 %, Sigma-Aldrich S.A.), tin octoate (92.5-100 %, Sigma-Aldrich S.A.) and benzyl alcohol (BnOH) (99.8 %, Sigma-Aldrich S.A.) were dried under vacuum before using them. L-lactide (Corbion) was purified in toluene and dried under vacuum before using it.

Methods

^{1}H and ^{13}C Nuclear Magnetic Resonance (NMR)

^{1}H and ^{13}C Nuclear Magnetic Resonance (NMR) spectroscopy was recorded in a Bruker Avance DPX 300 at 300.16 MHz and at 75.5 MHz of resonance frequency respectively, using deuterated chloroform ($CDCl_3$), deuterated dimethyl sulfoxide (DMSO) or deuterium oxide (D_2O) as solvent at room temperature. Experimental conditions were as follows: a) for ^{1}H NMR spectroscopy: 10 mg of sample; 3 s acquisition time; 1 s delay time; 8.5 µs pulse; spectral width 5000 Hz and 32 scans; b) for ^{13}C NMR spectroscopy: 40 mg; 3 s acquisition time; 4 s delay time; 5.5 µs pulse; spectral width 18800 Hz and more than 10000 scans.

Differential Scanning Calorimetry (DSC)

Differential scanning calorimetry (DSC) measurements were performed using a DSC8500 from Perkin Elmer, Inc. calibrated with indium and tin standards. The DSC scans were performed with approximately 5 mg of sample at a heating rate of 20 °C/min from -20 °C to 180 °C under a nitrogen flow rate of 20 mL/min.

Thermogravimetric analysis (TGA)

Thermogravimetric analysis (TGA) was carried out using a Q500 Thermogravimetric Analyzer from TA Instruments. Samples were heated from room temperature to 600 °C at a rate of 10 °C/min under a constant N_2 flow.

Matrix Assisted Laser Desorption Ionization - Time of Flight (MALDI-TOF) analysis

MALDI-TOF measurements were performed on a Bruker Autoflex Speed system (Bruker, Germany) instrument, equipped with a 355 nm NdYAG laser using chloroform as solvent and DCTB-NaTFA substrate.

Size Exclusion Chromatography (SEC)

Size Exclusion Chromatography (SEC) was performed in chloroform at 30 °C using a Waters chromatograph equipped with four 5 mm Waters columns (300 mm x 7.7 mm) connected in series with increasing pore sizes. Toluene was used as a marker and the calibration was done using polystyrene standards.

P_m value

P_m value is the probability of meso linkage between monomer units, and it was calculated from the methine region of the ^{13}C NMR spectrum: $[mmm] = P_m(P_m + 1)/2$; $[mmr] = P_m(1 - P_m)/2$; $[rmm] = P_m(1 - P_m)/2$; $[rmr] = (1 - P_m)^2/2$; $[mrm] = (1 - P_m)/2$[1–3].

X-ray diffraction

Single crystal diffractometer (SuperNova Cu) with four-circle goniometer, Kappa geometry and microfocus Cu source was used equipped with a large Atlas model two-dimensional CCD detector. The measurements were done at standards conditions, at 100 K.

Toxicity test

To assess the cytotoxicity of the developed materials, ISO/EN 10993 protocols were followed[4]. Extracts of the materials were obtained by incubating the polymer/the sample with complete medium (DMEM + 10 % FBS + 1 % P/S) at a ratio of sample mass to extraction medium of 200 mg/ml. They were incubated for 24 h at 37 °C in humidified atmosphere containing 5 % CO_2. HeLa cells were seeded at a concentration of 5000 cells/well on a 96 well-plate in complete medium. After 24 h in culture, complete media was replaced by previously filtered extracts. Cell cytotoxicity was then evaluated using the Alamar Blue assay after 24 h and 72 h.

Computational methods

The studies of complex formation, the reaction mechanism, and their corresponding kinetics were performed using the Gaussian 09 package, version D.01.[5] The ωB97XD[6,7] and the M06-2X[8] functionals were employed. The ωB97XD was applied to keep consistency with previous

theoretical studies conducted on similar systems[9] while the use of Minessota functional was dictated by its high performance for main group thermochemistry, kinetics, and non-covalent interactions[10], as well as by our own long experience in applying this functional in studying diverse bio-organic reactions[11–15] with many successful outcomes. The 6-31+G(d,p) basis set was employed for all atoms in both cases. To mimic the influence of the environment, the conductor-like polarizable continuum model (CPCM)[16–18] with a dielectric constant, $\varepsilon = 12.0$ was applied, which corresponds to the value of permittivity of the ethyl lactate experimentally determined in previous studies at $T = 358$ K[19]. To characterize optimized stationary points, diagonalized Hessian matrices were computed at the same levels of theory confirming that the localized structures correspond to minima (all positive eigenvalues) or transition states (one negative eigenvalue). Subsequently, the zero-point vibrational energy (ZPE) and the thermal vibrational corrections obtained at $T = 403$ K (the temperature employed in the experiments of the present study) were added to the electronic energy. The IRC method has been used to verify that the obtained transition states are related to the desired minima. A note of caution must be introduced at this point since, because the obtained reaction mechanisms correspond to a multistep process, the intermediate obtained from the IRC traced down from TS1 can be geometrically different from the INT1 obtained from TS2. However, the conformational differences between structures of the same state are not associated with a relevant energy cost. Moreover, r.d.s. is not affected since it corresponds to the same chemical transformation related to lactide ring-opening.

Ring-opening polymerization of L-lactide

The synthesis of polylactide polyester was performed by ROP of a cyclic ester, L-lactide. In a 5 mL vial 0.50 g ($3.47\ 10^{-3}$ mol) of L-lactide was placed with a magnetic bar, 5 mol % of organocatalyst ($1.73\ 10^{-3}$ mol) and 7.21 µL ($6.94\ 10^{-5}$ mol) of benzyl alcohol (DP 50).

The vial was then submerged into a pre-heated oil bath at 180 °C and the conversion was followed by ^{1}H NMR in deuterated chloroform. After reaction completion, the formed polylactide was let to cool down to room temperature naturally. For purification, the sample was dissolved in chloroform and precipitated in cold methanol. The resulted polyester was filtrated and dried under vacuum at RT for 24 h before its characterization.

^{1}H NMR (300 MHz, Chloroform-*d*) δ 5.16 (qd, *J* = 7.3,3.1 Hz, 1H), 1.57 (dd, *J* = 7.2, 1.5 Hz, 3H).**^{13}C NMR (75 MHz, Chloroform-*d*) δ** 170.70, 70.10, 17.73.

Ring-opening polymerization of ε-caprolactone

The ROP of ε-caprolactone was carried out following the same reaction conditions as for the polymerization of L-lactide. Employing 0.50 g (4.38 10^{-3} mol) of ε-caprolactone, 5 mol % of taurine (2.19 10^{-4} mol, 0.027 g) and 9.11 µL (8.76 10^{-5} mol) of benzyl alcohol (DP 50).

The vial was heated up at 180 °C and the evolution was followed by ^{1}H NMR in deuterated chloroform. After that, the same purification process as for the ROP of L-lactide was followed.

^{1}H NMR (300 MHz, Chloroform-*d*) δ 4.05 (t, *J* = 6.7 Hz, 2H), 2.30 (t, *J* = 7.5 Hz, 2H), 1.73 – 1.56 (m, 4H), 1.46 – 1.29 (m, 2H).

Ring-opening polymerization of trimethylene carbonate

The synthesis of polytrimethylene carbonate was performed in the same way as the ROP of L-lactide. In a 5 mL vial 0.50 g (4.90 10^{-3} mol) of trimethylene carbonate was placed with a magnetic bar, 5 mol % of taurine (1.73 10^{-3} mol, 0.031 g) and 10.2 µL (9.80 10^{-5} mol) of benzyl alcohol (DP 50).

The vial was heated up to 180 °C and the conversion was followed by ^{1}H NMR in deuterated chloroform. After 4 h of reaction, the polycarbonate was purified by the use of the same procedure as for the synthesis of polylactide.

^{1}H NMR (300 MHz, Chloroform-*d*) δ 4.24 (t, *J* = 6.2 Hz, 4H), 2.05 (p, *J* = 6.2 Hz, 2H).

Characterization Data and Results

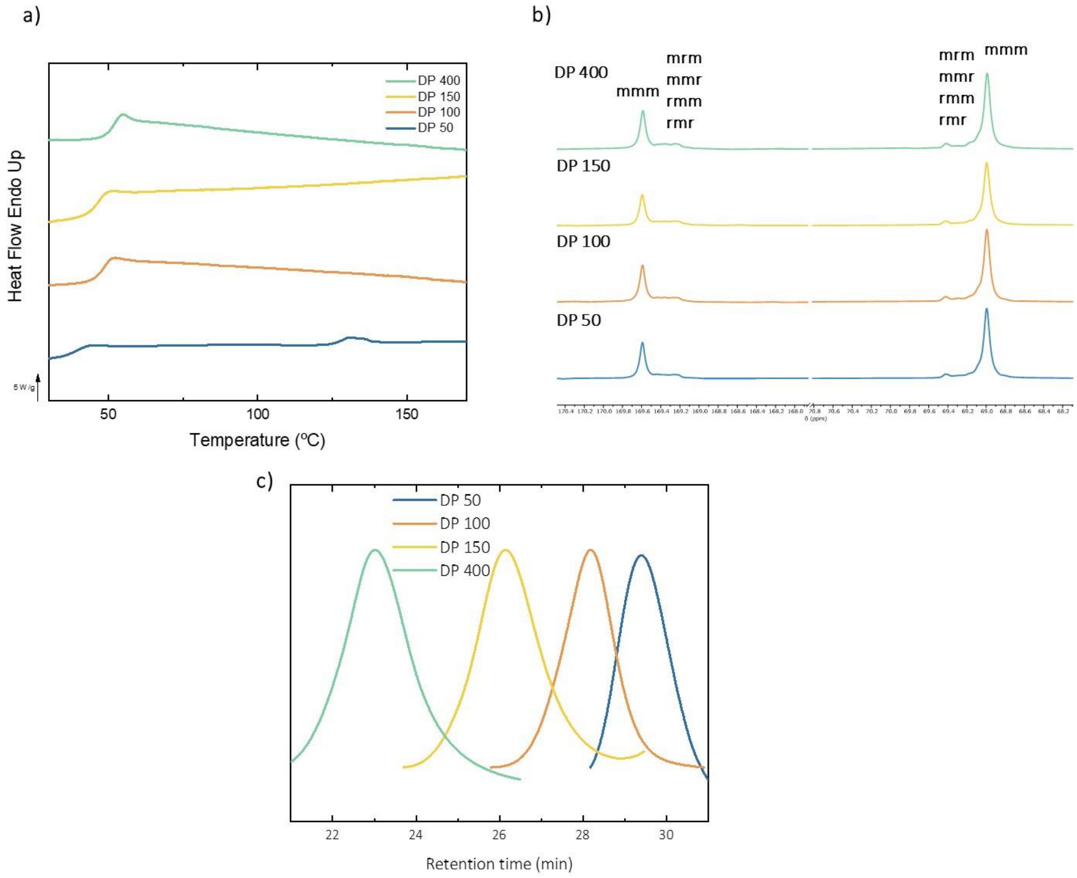

Figure S1. a) DSC analysis, b) ^{13}C NMR spectra and c) SEC curves of poly(L-lactide) of 50, 100, 150 and 400 polymerization degrees.

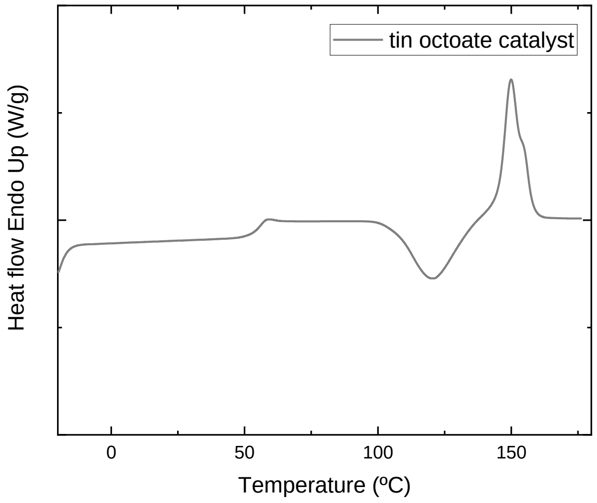

Figure S2. DSC analysis of PLA synthesized with tin octoate.

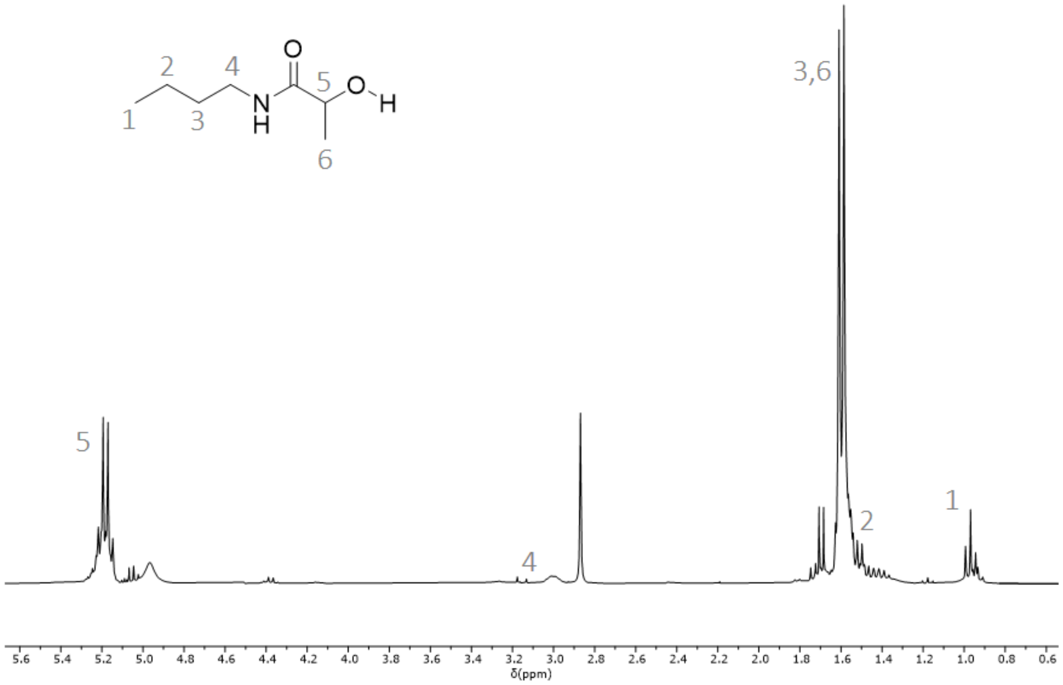

Figure S3. ^{1}H NMR spectrum of the ROP of L-lactide initiated by MSA:butylamine salt.

a)

entry	catalyst	time (h)	conv. (%)	$M_{n,\,theo}$ (g mol^{-1})	$M_{n,\,SEC}$ (g mol^{-1})^a	Đ
1	Taurine	7	98	14200	14100	1.1
3	L-Proline	4	100	14500	7300	1.5
5	Creatine	3	99	14400	2500	1.6

[a] Determined by SEC in THF with polystyrene standards and correction factors.

b)

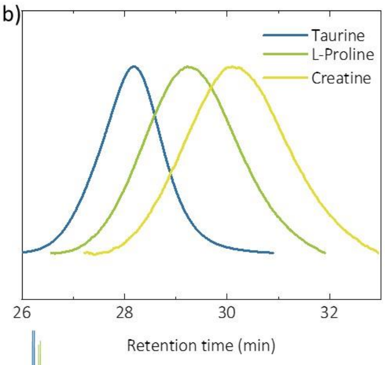

c)

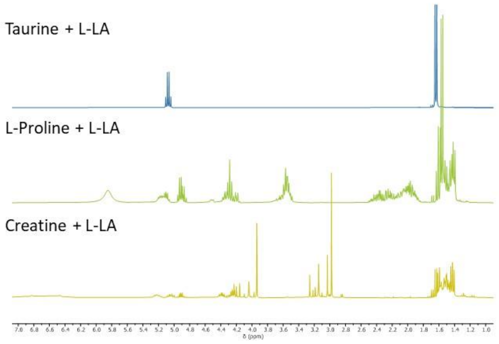

Figure S4. a) Conditions and results for the ROP of L-Lactide with unimolecular acid-base naturally occurring catalyst, b) SEC curves and c) ^{1}H NMR spectra of the catalysts mixed with L-lactide.

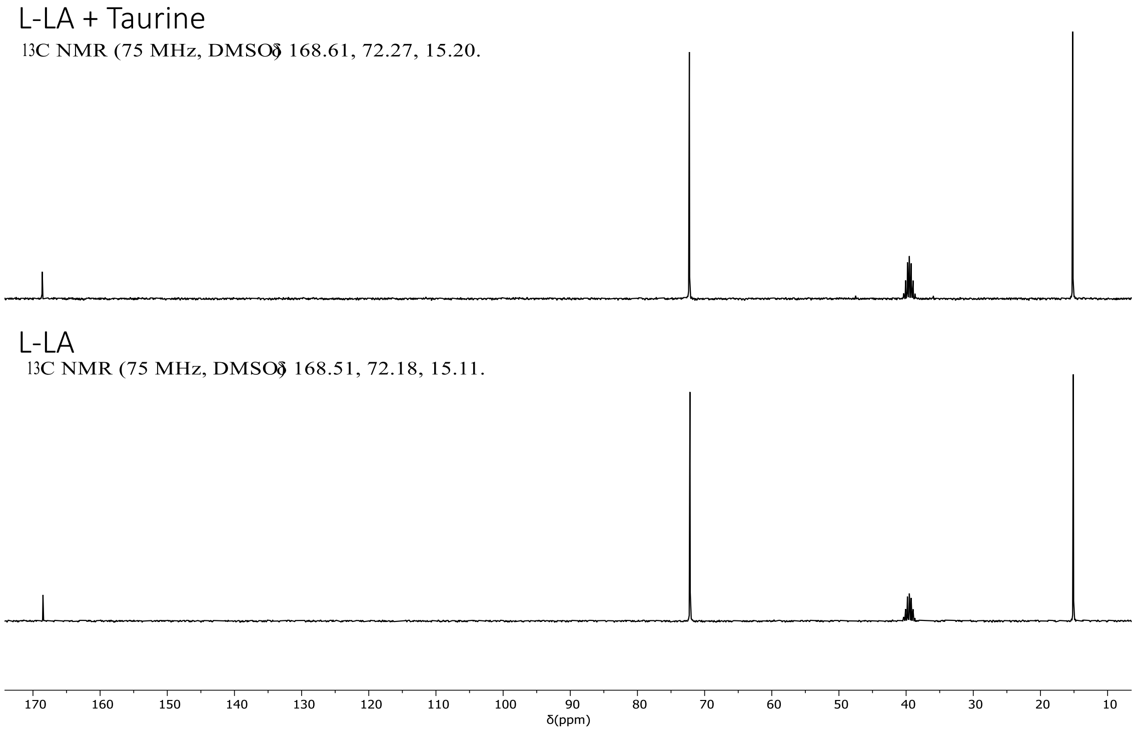

Figure S5. ^{13}C NMR spectra of L-LA in contact with taurine (L-LA + Taurine) and L-LA.

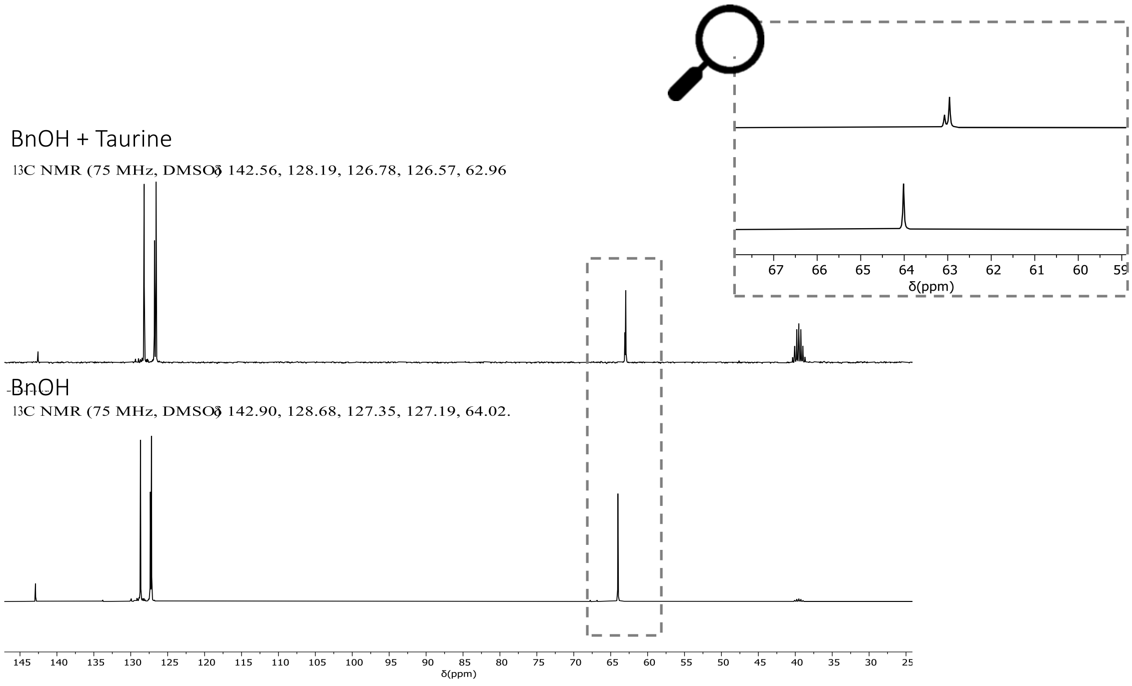

Figure S6. ^{13}C NMR spectra of BnOH in contact with taurine (BnOH + Taurine) and BnOH.

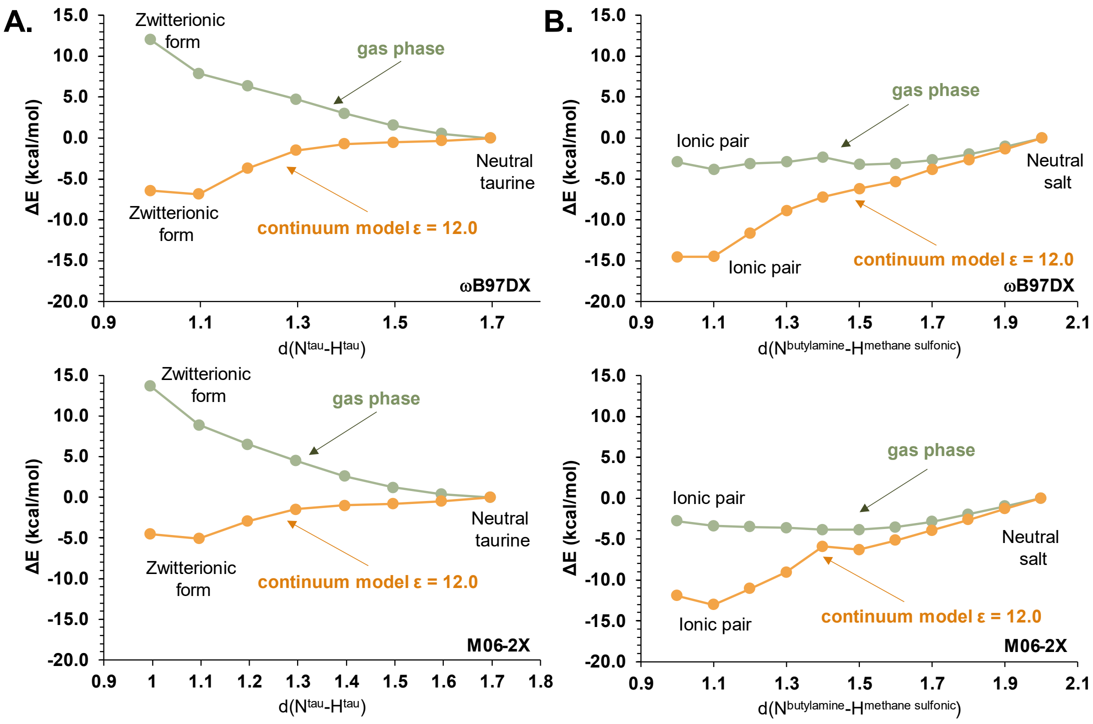

Figure S7. Potential energy surface explored with ωB97XD and M06-2X functionals for A. intramolecular proton transfer from amino (-NH_2) to sulfone (-SO_3) group in taurine and B. intermolecular proton transfer from amino (-NH_2) group of butylamine to sulfone (-SO_3) group of methane sulfonic acid. Potential energies computed in gas phase and with the continuum solvent model are represented by green and orange circles, respectively.

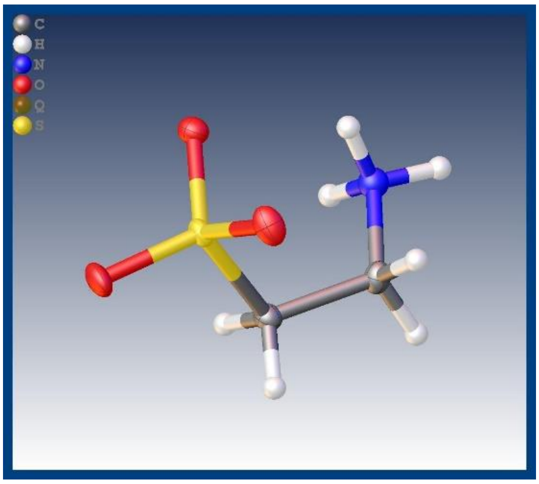

Figure S8. Structure of taurine confirmed by X-ray analysis.

Ring-opening polymerization (ROP) process of L-lactide (L-LA) with benzyl alcohol (BnOH) and butylamine ($BuNH_2$). Uncatalyzed reaction

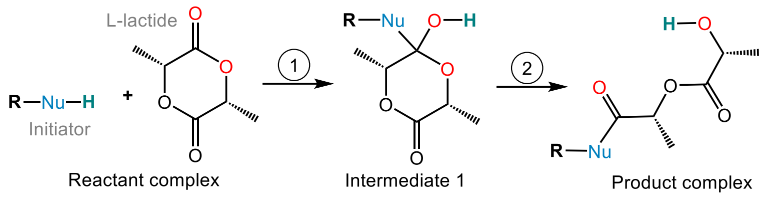

Figure S9. Proposed two-step mechanism for the <u>uncatalyzed</u> initiation step of the ring-opening polymerization (ROP) of L-lactide (L-LA) with benzyl alcohol (BnOH) and butylamine ($BuNH_2$) as initiators. Depending on the structure of the initiator, the nucleophile (Nu) corresponds to the oxygen and nitrogen atom, and the -R substituent is the benzyl or butyl group, respectively.

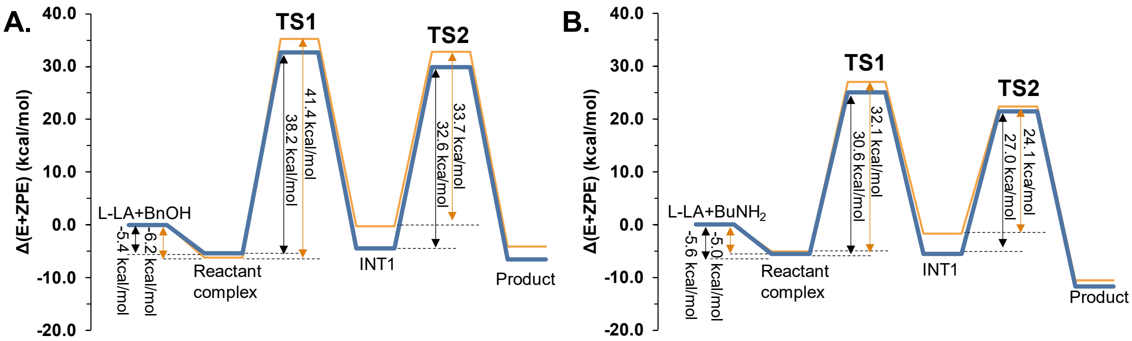

Figure S10. Energy profiles of the <u>uncatalyzed</u> initiation step of the ring-opening polymerization of L-lactide(L-LA) with **A.** benzyl alcohol (BnOH) and **B.** butylamine ($BuNH_2$) as initiators computed at ωB97XD (in orange) and M06-2X (in blue) level at T = 403 K.

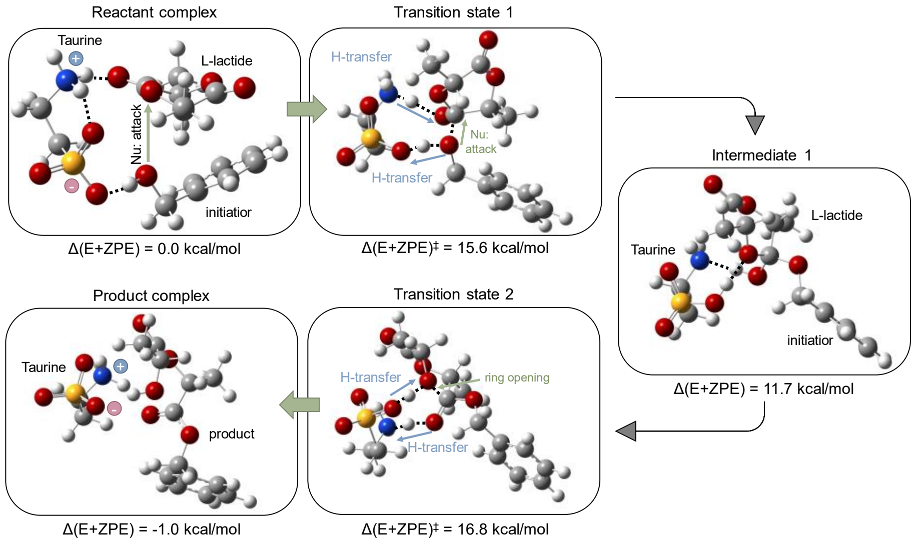

Figure S11. Structure and relative energies of optimized and characterized stationary points along the taurine-assisted initiation step of ROP of L-LA and BnOH playing the role of the initiator at M06-2X level.

<u>The ROP of the L-LA reaction catalyzed by salt</u>

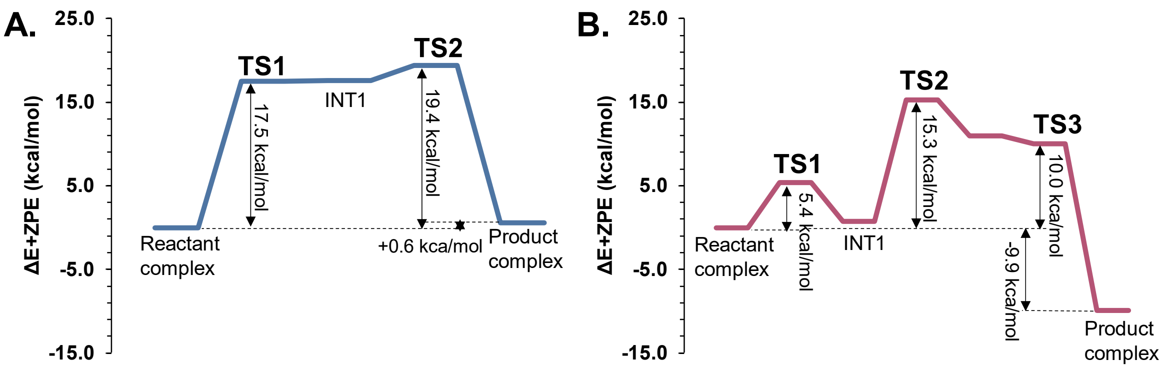

Figure S12. Energy profiles of the $BuNH_2$:MSA assisted process of ring-opening polymerization of L-lactide(L-LA) with **A.** benzyl alcohol (BnOH) and **B.** butylamine ($BuNH_2$) computed at M06-2X level at T = 403 K.

According to the mechanistic studies, the ROP of L-LA assisted by ionic pair of $BuNH_2$:MSA salt with neutral $BuNH_2$ playing the role of initiator takes place in three steps. The two first steps correspond to the step-wise process in which the amino group of $BuNH_2$ is initially covalently attached to the carbonyl carbon ($C=O^{L\text{-}La}$) of L-LA resulting in a zwitterionic intermediate (INT1), which is then converted to the neutral intermediate (INT2) by double proton transfer, one from the nitrogen of amino group of the $BuNH_2$ initiator to the negatively charged oxygen of MSA and one from the amino group of positively charged $BuNH_2$ from $BuNH_2$:MSA complex to carbonyl oxygen of L-LA. The third step of the reaction is equivalent to TS2 of the ROP assisted by Tau and consists of the return transfer of the proton from carbonyl oxygen of L-LA to the amino group of $BuNH_2$ of salt, transfer of the proton from MSA to the oxygen of the L-LA ring and finally the L-LA ring-opening.

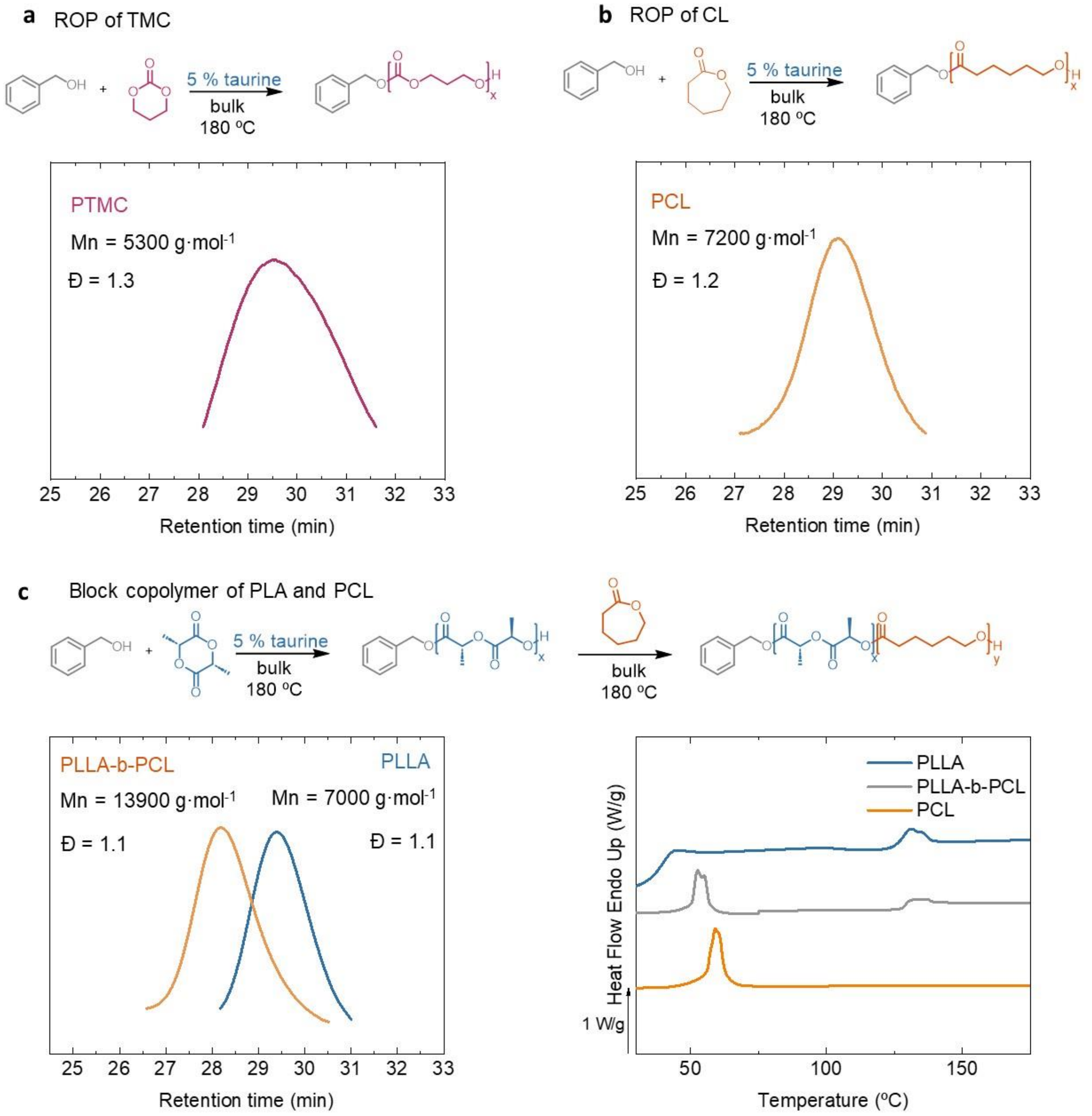

Figure S13. ROP of a) TMC, b) CL and c) synthesis of PLA-b-PCL block copolymer and their SEC and DSC results.

Table S1. Key distances (in Å) of optimized and characterized stationary points along the uncatalyzed processes of the ring-opening polymerization of L-lactide(L-LA) with benzyl alcohol (BnOH) and butylamine ($BuNH_2$) as initiators computed at ωB97XD and M06-2X level at T = 403 K.

	ωB97XD										
	benzyl alcohol (BnOH)						butylamine ($BuNH_2$)				
Distance	RC	TS1	INT1	TS2	PC	Distance	RC	TS1	INT1	TS2	PC
O^{BnOH}-H^{BnOH}	0.96	1.18	2.41/3.04	2.70	3.65	N^{BuNH2}-H^{BuNH2}	1.02	1.25	2.42/3.08	2.81	3.60
H^{BnOH}-O=C^{L-LA}	3.42	1.28	0.96/0.96	1.22	3.06	H^{BuNH2}-O=C^{L-LA}	3.44	1.30	0.97/0.96	1.02	2.95
O=C^{L-LA}-C=O^{L-LA}	1.21	1.31	1.39/1.39	1.31	1.21	O=C^{L-LA}-C=O^{L-LA}	1.21	1.35	1.40/1.39	1.32	1.23
O^{BnOH}-C=O^{L-LA}	3.08	1.67	1.40/1.38	1.32	1.33	N^{BuNH2}-C=O^{L-LA}	3.14	1.54	1.44/1.42	1.31	1.35
C=O^{L-LA}-$O^{L-LA(ring)}$	1.34	1.36	1.40/1.41	1.79	2.76	C=O^{L-LA}-$O^{L-LA(ring)}$	1.34	1.41	1.41/1.44	2.17	2.79
H^{BnOH}-$O^{L-LA(ring)}$	3.71	2.61	3.02/2.20	1.23	0.96	H^{BuNH2}-$O^{L-LA(ring)}$	3.72	2.62	2.47/2.22	1.59	0.96
	M06-2X										
	benzyl alcohol (BnOH)						butylamine ($BuNH_2$)				

Distance	RC	TS1	INT1	TS2	PC	Distance	RC	TS1	INT1	TS2	PC
O^{BnOH}-H^{BnOH}	0.97	1.16	2.62/3.05	2.69	3.60	N^{BuNH2}-H^{BuNH2}	1.02	1.24	2.42/3.08	2.79	3.44
H^{BnOH}-O=C^{L-LA}	3.36	1.31	0.97/0.97	1.24	2.92	H^{BuNH2}-O=C^{L-LA}	3.22	1.32	0.97/0.97	1.04	2.34
O=C^{L-LA}-C=O^{L-LA}	1.20	1.31	1.38/1.39	1.31	1.21	O=C^{L-LA}-C=O^{L-LA}	1.20	1.34	1.40/1.39	1.32	1.23
O^{BnOH}-C=O^{L-LA}	2.79	1.64	1.40/1.38	1.33	1.33	N^{BuNH2}-C=O^{L-LA}	2.89	1.54	1.44/1.43	1.31	1.34
C=O^{L-LA}-$O^{L-LA(ring)}$	1.34	1.37	1.40/1.41	1.76	2.69	C=O^{L-LA}-$O^{L-LA(ring)}$	1.34	1.41	1.41/1.43	2.10	2.75
H^{BnOH}-$O^{L-LA(ring)}$	3.65	2.60	2.34/2.21	1.21	0.97	H^{BuNH2}-$O^{L-LA(ring)}$	3.67	2.64	2.47/2.23	1.53	0.97

Table S2. Mulliken charges (in a.u.) of key atoms in optimized and characterized stationary points along the uncatalyzed processes of the ring-opening polymerization of L-lactide(L-LA) with benzyl alcohol (BnOH) and butylamine ($BuNH_2$) as initiators computed at ωB97XD and M06-2X level at T = 403 K.

	ωB97XD										
	benzyl alcohol (BnOH)						butylamine ($BuNH_2$)				
Distance	RC	TS1	INT1	TS2	PC	Distance	RC	TS1	INT1	TS2	PC
O^{BnOH}	-0.379	-0.416	-0.277/-0.242	-0.188	-0.220	N^{BuNH2}	-0.783	-0.447	-0.327/-0.277	-0.218	-0.278
H^{BnOH}	0.435	0.444	0.387/0.404	0.457	0.400	H^{BuNH2}	0.325	0.415	0.398/0.396	0.436	0.398
O=C^{L-LA}	-0.612	-0.596	-0.419/-0.525	-0.594	-0.453	O=C^{L-LA}	-0.473	-0.702	-0.525/-0.460	-0.482	-0.565
C=O^{L-LA}	-0.165	-0.247	-0.474/-0.281	-0.110	0.047	C=O^{L-LA}	0.384	0.026	-0.307/-0.728	0.093	0.213
$O^{L-LA(ring)}$	-0.325	-0.303	-0.277/-0.397	0.494	-0.525	$O^{L-LA(ring)}$	-0.276	-0.363	-0.343/-0.352	-0.720	-0.527
	M06-2X										
	benzyl alcohol (BnOH)						butylamine ($BuNH_2$)				
Distance	RC	TS1	INT1	TS2	PC	Distance	RC	TS1	INT1	TS2	PC
O^{BnOH}	-0.607	-0.424	-0.246/-0.271	-0.225	-0.256	NBuNH2	-0.868	-0.548	-0.385/-0.346	-0.288	-0.360
H^{BnOH}	0.421	0.459	0.403/0.413	0.465	0.413	H^{BuNH2}	0.336	0.438	0.408/0.406	0.456	0.408
O=C^{L-LA}	-0.428	-0.602	-0.388/-0.485	-0.574	-0.412	O=C^{L-LA}	-0.450	-0.689	-0.519/-0.470	-0.503	-0.598
C=O^{L-LA}	0.199	-0.160	-0.441/-0.325	-0.148	-0.059	C=O^{L-LA}	0.417	0.009	-0.415/-0.626	0.058	0.334
$O^{L-LA(ring)}$	-0.293	-0.329	-0.393/-0.402	-0.498	-0.555	$O^{L-LA(ring)}$	-0.280	-0.388	-0.349/-0.358	-0.687	-0.524

Table S3. Computed energy (ΔE+ZPE) profiles (in kcal/mol) for uncatalyzed processes of ring-opening polymerization of L-lactide(L-LA) with benzyl alcohol (BnOH) and butylamine ($BuNH_2$) as initiators computed at ωB97XD and M06-2X level at T = 403 K.

State	benzyl alcohol (BnOH)		butylamine ($BuNH_2$)	
	ωB97XD	M06-2X	ωB97XD	M06-2X
L-LA + initiator	0.00	0.00	0.00	0.00
RC	-6.16	-5.44	-5.01	-5.56
TS1	35.28	32.73	27.10	25.05
INT1	-0.29	-4.43	-1.66	-5.53
TS2	32.83	29.89	22.44	21.52
Opened-L-LA	-4.09	-6.52	-10.50	-11.66

The ROP of the L-LA reaction catalyzed by taurine

The established H-bond interaction between reactants and catalyst ensures the reactive orientation required for the first step of the reaction, i.e. the nucleophilic attack, by bringing close together the nucleophilic group and electrophilic center (at a distance ($d_{Nu:-C=O}$) of 3.32 and 3.11 Å, and a Bürgi-Dunitz[14,20] (α_{BD}) angle of 109.7° and 104.8° defined as an angle between the nucleophile, the carbonyl atom, and the carbonyl oxygen, for structures optimized at ωB97XD and M06-2X level, respectively). The value of 105 ± 5° determined by Bürgi et al. for small-molecule

substrates is the angle that ensures a reliable position for the nucleophile to attack[20]. Anyway, the starting orientation of the reactants in the uncatalyzed process is characterized by similar short distance $d_{Nu:-C=O}$ of 3.08 and 2.79 Å and favorable α_{BD} angle of 101.7° and 99.5° determined at ωB97XD and M06-2X level, respectively, suggesting that the relative position of both reactants is not responsible in this case for the observed meaningful reduction of the energy barrier for this step in the Tau assisted process.

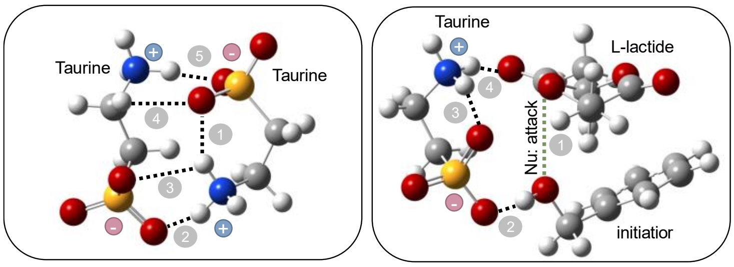

(Tau_2)
$\Delta E+ZPE^{(\omega B97DX)}$ = -13.9 kcal/mol
$\Delta E+ZPE^{(M06-2X)}$ = -14.8 kcal/mol

H-bond distance	wB97DX	M06-2X
d1	1.90	1.94
d2	1.78	1.86
d3	2.51	2.28
d4	2.57	2.42
d5	1.70	1.71

Tau:L-LA:BnOH
$\Delta E+ZPE^{(\omega B97DX)}$ = -22.3 kcal/mol
$\Delta E+ZPE^{(M06-2X)}$ = -20.8 kcal/mol

H-bond distance	wB97DX	M06-2X
d1	3.32	3.11
d2	1.77	1.77
d3	1.85	1.87
d4	1.83	1.90

Figure S14. Optimized structures of the dimer of taurine (Tau_2) and taurine- L-lactide- benzyl alcohol complex (Tau:L-LA:BnOH) and energies of formation ($\Delta E+ZPE_f$) computed at ωB97XD and M06-2X level at T = 403 K.

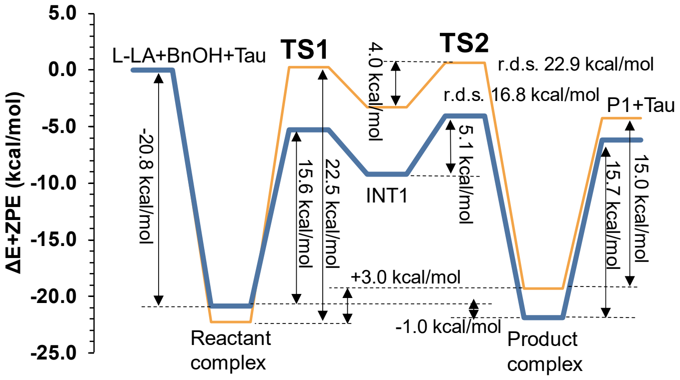

Figure S15. Potential energy profiles with ZPE corrections for taurine-assisted ROP of L-lactide(L-LA) with benzyl alcohol (BnOH) initiator computed at ωB97XD (in orange) and M06-2X (in blue) level at T = 403 K.

Our calculations show that both DFT functionals provide the same molecular mechanism of taurine-assisted ROP of L-LA, being step two the r.d.s., and with very similar geometries of optimized stationary structures. Nevertheless, the values of the computed energy barriers differ significantly from each other. In the case of the ωB97XD functional, the computed energy of activation was 22.9 kcal/mol while at M06-2X it was 16.8 kcal/mol. Considering the value of

experimentally determined activation energy of 17.4 kcal/mol calculated for this process using Arrhenius plots and in the framework of the Transition State Theory, it was concluded that M06-2X provides results in better agreement with experiments. Moreover, only in the case of results computed at M06-2X the final product complex was found to be thermodynamically more stable (by 1.1 kcal/mol) than the reactant complex. This was not the case for ωB97XD. Therefore, the remaining computational simulations and analyses presented in this work were conducted based on results obtained with the Minnesota functional.

Table S4. Computed energy profiles (in kcal/mol) for taurine-assisted processes of ring-opening polymerization of L-lactide (L-LA) with benzyl alcohol (BnOH) initiators computed at ωB97XD and M06-2X level.

State	ωB97XD	M06-2X
	Taurine-assisted	Taurine-assisted
L-LA+BnOH+catalyst	0.00	0.00
RC	-22.28	-20.84
TS1	0.24	-5.28
INT1	-3.28	-9.18
TS2	0.67	-4.04
PC	-19.31	-21.85
P + catalyst	-4.28	-6.18

Table S5. Key distances (in Å) of optimized and characterized stationary points along ROP progress in the presence of taurine molecule as a catalyst and BnOH playing the role of initiator computed at ωB97XD and M06-2X level at T = 403 K.

	ωB97XD					M06-2X				
Distance	RC	TS1	INT1	TS2	PC	RC	TS1	INT1	TS2	PC
O^{BnOH}-H^{BnOH}	0.98	1.28	1.52/3.38	3.22	3.82	0.98	1.21	1.59/3.31	2.80	3.75
H^{BnOH}-O^{Tau}	1.77	1.13	1.03/1.00	1.15	1.80	1.77	1.19	1.01/1.01	1.21	1.83
N^{Tau}-H^{Tau}	1.04	1.09	1.13/1.67	1.08	1.04	1.03	1.11	1.60/1.62	1.10	1.03
H^{Tau}-O=C^{L-LA}	1.83	1.54	1.41/1.01	1.53	1.83	1.90	1.49	1.03/1.02	1.48	1.86
O=C^{L-LA}-C=O^{L-LA}	1.22	1.28	1.50/1.36	1.28	1.22	1.21	1.28	1.35/1.36	1.27	1.22
O^{BnOH}-C=O^{L-LA}	3.32	1.60	1.30/1.48	1.38	1.32	2.80	1.60	1.45/1.38	1.38	1.32
C=O^{L-LA}-$O^{L-LA(ring)}$	1.32	1.40	1.42/1.44	1.65	2.83	1.33	1.40	1.39/1.44	1.68	2.75
H^{BnOH}-$O^{L-LA(ring)}$	3.56	2.45	2.51/1.65	1.26	0.98	3.02	2.39	2.53/1.59	1.19	0.98

Table S6. Mulliken charges (in a.u.) on key atoms of optimized and characterized stationary points along ROP progress in the presence of taurine molecule as a catalyst and BnOH playing the role of initiator computed at ωB97XD and M06-2X level at T = 403 K.

	ωB97XD					M06-2X				
Atoms	RC	TS1	INT1	TS2	PC	RC	TS1	INT1	TS2	PC
O^{BnOH}	-0.619	-0.541	-0.471/-0.191	-0.168	-0.162	-0.659	-0.589	-0.505/-0.212	-0.136	-0.185
H^{BnOH}	0.441	0.609	0.555/0.523	0.600	0.430	0.476	0.624	0.558/0.557	0.617	0.454
O^{Tau}	-0.704	-0.705	-0.640/-0.662	-0.723	-0.708	-0.690	-0.679	-0.620/-0.662	-0.681	-0.681
N^{Tau}	-0.677	-0.680	-0.719/-0.773	-0.659	-0.658	-0.760	-0.779	-0.864/-0.858	-0.812	-0.757
H^{Tau}	0.436	0.480	0.505/ 0.502	0.484	0.449	0.454	0.494	0.513/0.516	0.502	0.480
O=C^{L-LA}	-0.456	-0.529	-0.580/-0.464	-0.571	-0.487	-0.315	-0.454	-0.435/-0.447	-0.447	-0.455
C=O^{L-LA}	0.235	-0.747	-0.794/-2.211	-1.746	-0.408	0.030	-0.942	-0.920/-1.666	-1.337	-0.539
$O^{L-LA(ring)}$	-0.236	-0.267	-0.273/-0.328	-0.442	-0.533	-0.295	-0.309	-0.284/-0.473	-0.639	-0.536

Table S7. Key distances (in Å) of optimized and characterized stationary points along ROP progress in the presence of butylamine-methane sulfonic acid salt as a catalyst and BnOH/$BuNH_2$ playing the role of initiator computed at M06-2X level at T = 403 K.

	BnOH initiator				
Distance	RC	TS1	INT1	TS2	PC
O^{BnOH}-H^{BnOH}	0.99	1.21	1.48 / 3.43	3.38	2.95
H^{BnOH}-O^{salt}	1.69	1.18	1.03 / 1.08	1.12	1.79
N^{salt}-H^{salt}	1.03	1.09	1.55 / 1.09	1.08	1.03
H^{salt}-O=C^{L-LA}	2.46	1.54	1.05 / 1.53	1.56	2.08
O=C^{L-LA}-C=O^{L-LA}	1.21	1.27	1.34 / 1.30	1.29	1.21
O^{BnOH}-C=O^{L-LA}	2.83	1.59	1.45 / 1.40	1.39	1.32
C=O^{L-LA}-$O^{L-LA(ring)}$	1.34	1.41	1.40 / 1.54	1.57	2.66
H^{BnOH}-$O^{L-LA(ring)}$	2.97	2.41	2.45 / 1.36	1.30	0.98

	$BuNH_2$ initiator						
Distance	RC	TS1	INT1	TS2	INT2	TS3	PC
N^{BuNH2}-H^{BuNH2}	1.02	1.02	1.03/1.05	2.28	2.52 / 2.83	2.62	2.92
H^{BuNH2}-O^{salt}	2.25	2.33	2.10/1.76	0.99	1.02 / 1.03	1.24	1.78
N^{salt}-H^{salt}	1.03	1.05	1.11/1.52	1.67	1.65 / 1.63	1.51	1.05
H^{salt}-O=C^{L-LA}	1.89	1.77	1.47/1.07	1.02	1.02 / 1.02	1.07	1.68
O=C^{L-LA}-C=O^{L-LA}	1.21	1.24	1.30/1.35	1.38	1.38 / 1.37	1.34	1.24
N^{BuNH2}-C=O^{L-LA}	2.79	1.98	1.58/1.52	1.44	1.44 / 1.42	1.39	1.33
C=O^{L-LA}-$O^{L-LA(ring)}$	1.33	1.37	1.42/1.41	1.44	1.45 / 1.47	1.60	2.66
H^{BnOH}-$O^{L-LA(ring)}$	3.30	2.86	2.50/2.39	1.85	1.56 / 1.51	1.16	0.98

Table S8. Mulliken charges (in a.u.) on key atoms optimized and characterized stationary points along ROP progress in the presence of butylamine-methane sulfonic acid salt as a catalyst and BnOH/$BuNH_2$ playing the role of initiator computed at M06-2X level at T = 403 K.

	BnOH initiator				
Atoms	RC	TS1	INT1	TS2	PC
O^{BnOH}	-0.716	-0.558	-0.544 /-0.193	-0.187	-0.180
H^{BnOH}	0.496	0.616	0.580 / 0.593	0.598	0.457
O^{salt}	-0.750	-0.642	-0.558 /-0.828	-0.858	-0.951
N^{salt}	-0.852	-0.747	-0.836 /-0.882	-0.873	-0.818
H^{salt}	0.477	0.470	0.510 / 0.526	0.522	0.491
O=C^{L-LA}	-0.397	-0.444	-0.417 /-0.550	-0.533	-0.453
C=O^{L-LA}	0.269	-0.608	-0.616 /-2.000	-1.937	-0.326
$O^{L-LA(ring)}$	-0.251	-0.311	-0.285 /-0.460	-0.473	-0.498

	$BuNH_2$ initiator						
Atoms	RC	TS1	INT1	TS2	INT2	TS3	PC
N^{BuNH2}	-0.971	-0.831	-0.762 /-0.988	-0.457	-0.440 /-0.377	-0.412	-0.332
H^{BuNH2}	0.359	0.426	0.526 / 0.537	0.535	0.585 / 0.561	0.367	0.482
O^{salt}	-0.600	-0.608	-0.824 /-0.815	-0.601	-0.652 /-0.650	-0.758	-0.780
N^{salt}	-0.786	-0.773	-0.758 /-0.906	-0.890	-0.896 /-0.914	-0.940	-0.813

H^{salt}	0.448	0.461	0.487 / 0.527	0.514	0.515 / 0.507	0.515	0.479
$O{=}C^{L\text{-}LA}$	-0.343	-0.353	-0.445 /-0.469	-0.458	-0.450 /-0.508	-0.455	-0.397
$C{=}O^{L\text{-}LA}$	0.120	-0.807	-1.414 /-0.463	-0.722	-0.535 /-1.119	-1.159	-0.520
$O^{L\text{-}LA(ring)}$	-0.218	-0.208	-0.213 /-0.208	-0.418	-0.506 /-0.245	-0.343	-0.494

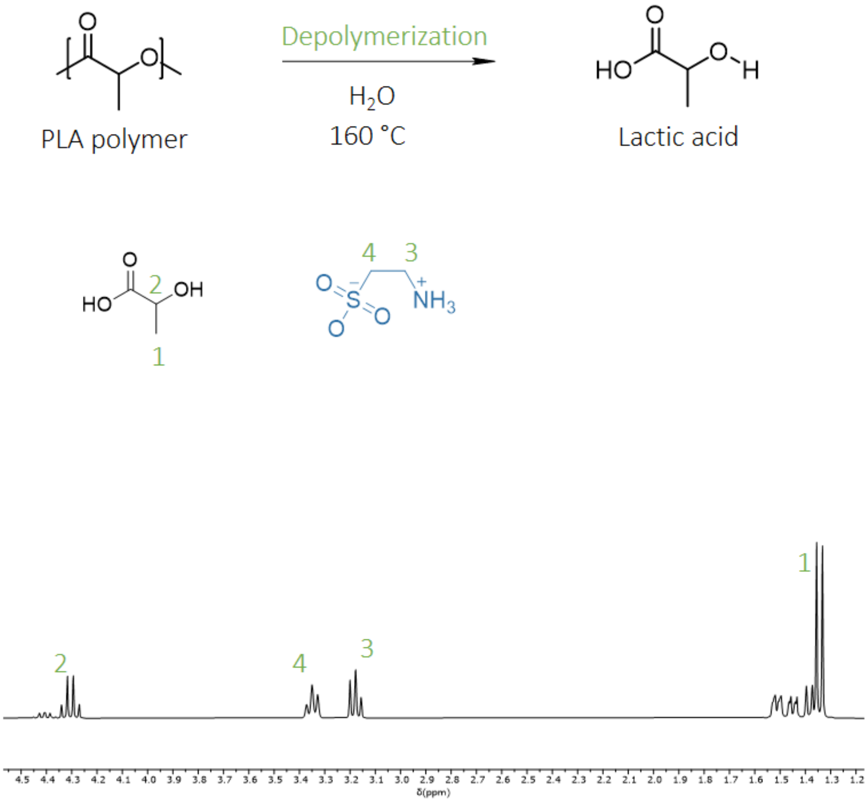

Figure S16. Hydrolytic depolymerization of PLA in presence of the catalyst taurine.

References

1 M. J. Stanford and A. P. Dove, *Chem. Soc. Rev.*, 2010, **39**, 486–494.

2 J. Kasperczyk and M. Bero, *Polymer*, 2000, **41**, 391–395.

3 J. Coudane, C. Ustariz-Peyret, G. Schwach and M. Vert, *J Polym Sci A Polym Chem*, 1997, **35**, 1651–1658.

4 *(ISO 10993-122012). Madrid AENOR, 2012*.

5 D. J. F. M.J. Frisch, G.W. Trucks, H.B. Schlegel, G.E. Scuseria, M.A. Robb, J.R. Cheeseman, G. Scalmani, V. Barone, B. Mennucci, G.A. Petersson, H. Nakatsuji, M. Caricato, X. Li, H.P. Hratchian, A.F. Izmaylov, J. Bloino, G. Zheng, J.L. Sonnenberg, M. Hada, M. Ehar, *No Title*, Wallingford, Gaussian 0., 2010.

6 J. Da Chai and M. Head-Gordon, *J. Chem. Phys.*, 2008, **128**, 084106.

7 J. Da Chai and M. Head-Gordon, *Phys. Chem. Chem. Phys.*, 2008, **10**, 6615–6620.

8 Y. Zhao, N. E. Schultz and D. G. Truhlar, *J. Chem. Theory Comput.*, 2006, **2**, 364–382.

9 A. Basterretxea, E. Gabirondo, C. Jehanno, H. Zhu, O. Coulembier, D. Mecerreyes and H. Sardon, *Macromolecules*, 2021, **54**, 6214–6225.

10 N. Mardirossian and M. Head-Gordon, *Mol. Phys.*, 2017, **115**, 2315–2372.

11 V. Świderek, K.; Velasco-Lozano, S.; Galmés, M. À.; Olazabal, I.; Sardon, H.; López-Gallego, F.; Moliner, *Nat. Commun.*, 2023, **14**, 3556.

12 A. Krzemińska, V. Moliner and K. Świderek, *J. Am. Chem. Soc.*, 2016, **138**, 16283–16298.

13 K. Świderek and V. Moliner, *Chem. Sci.*, 2020, **11**, 10626–10630.

14 N. Serrano-Aparicio, V. Moliner and K. Swiderek, *ACS Catal.*, 2021, **11**, 3575–3589.

15 M. i. Galmés, A. R. Nödling, K. He, L. Y. P. Luk, K. Świderek and V. Moliner, *Chem. Sci.*, 2022, **13**, 4779–4787.

16 S. Miertuš, E. Scrocco and J. Tomasi, *Chem. Phys.*, 1981, **55**, 117–129.

17 S. Miertuš̃ and J. Tomasi, *Chem. Phys.*, 1982, **65**, 239–245.

18 J. L. Pascual-ahuir, E. Silla and I. Tuñon, *J. Comput. Chem.*, 1994, **15**, 1127–1138.

19 S. Aparicio, S. Halajian, R. Alcalde, B. García and J. M. Leal, *Chem. Phys. Lett.*, 2008, **454**, 49–55.

20 H. B. Biirgi, J. D. Dunitz and E. Shefter, *J. Am. Chem. Soc.*, 1973, **95**, 5065–5067.
